# Oxidative Cysteine Post Translational Modifications Drive the Redox Code Underlying Neurodegeneration and Amyotrophic Lateral Sclerosis

**DOI:** 10.3390/antiox13080883

**Published:** 2024-07-23

**Authors:** Anna Percio, Michela Cicchinelli, Domiziana Masci, Mariagrazia Summo, Andrea Urbani, Viviana Greco

**Affiliations:** 1 Department of Basic Biotechnological Sciences, Intensivological and Perioperative Clinics, Università Cattolica del Sacro Cuore, 00168 Rome, Italy; anna.percio@unicatt.it (A.P.); michela.cicchinelli@unicatt.it (M.C.); domiziana.masci@unicatt.it (D.M.); mariagrazia.summo01@icatt.it (M.S.); andrea.urbani@unicatt.it (A.U.); 2 Department of Laboratory Diagnostic and Infectious Diseases, Unity of Chemistry, Biochemistry and Clinical Molecular Biology, Fondazione Policlinico Universitario Agostino Gemelli-IRCCS, 00168 Rome, Italy

**Keywords:** amyotrophic lateral sclerosis, cysteine PTMs, mitochondria, neurodegeneration, proteome, redox dysregulation

## Abstract

Redox dysregulation, an imbalance between oxidants and antioxidants, is crucial in the pathogenesis of various neurodegenerative diseases. Within this context, the “redoxome” encompasses the network of redox molecules collaborating to maintain cellular redox balance and signaling. Among these, cysteine-sensitive proteins are fundamental for this homeostasis. Due to their reactive thiol groups, cysteine (Cys) residues are particularly susceptible to oxidative post-translational modifications (PTMs) induced by free radicals (reactive oxygen, nitrogen, and sulfur species) which profoundly affect protein functions. Cys-PTMs, forming what is referred to as “cysteinet” in the redox proteome, are essential for redox signaling in both physiological and pathological conditions, including neurodegeneration. Such modifications significantly influence protein misfolding and aggregation, key hallmarks of neurodegenerative diseases such as Alzheimer’s, Parkinson’s, and notably, amyotrophic lateral sclerosis (ALS). This review aims to explore the complex landscape of cysteine PTMs in the cellular redox environment, elucidating their impact on neurodegeneration at protein level. By investigating specific cysteine-sensitive proteins and the regulatory networks involved, particular emphasis is placed on the link between redox dysregulation and ALS, highlighting this pathology as a prime example of a neurodegenerative disease wherein such redox dysregulation is a distinct hallmark.

## 1. Introduction

Redox homeostasis, the intricate balance between oxidation and reduction processes, is fundamental for cellular survival and function. This equilibrium governs key cellular mechanisms and is regulated by a dynamic network known as “redoxome”. Within the redoxome, a variety of redox-active molecules, including antioxidants, enzymes, and regulatory proteins, collaborate to maintain cellular redox balance and signaling.

Disruptions in this equilibrium, termed redox dysregulation, significantly contribute to various diseases. Oxidative stress (OS), resulting from an imbalance between the production of reactive species (including reactive oxygen (ROS), nitrogen (RNS), and sulfur (RSS) species) and antioxidant defenses leads to cellular damage and dysfunction. This is particularly relevant in neurodegenerative disorders (NDDs) such as Alzheimer’s disease (AD), Parkinson’s disease (PD), frontotemporal dementia (FTD), and amyotrophic lateral sclerosis (ALS). In particular, redox dysregulation has been recognized as a key driver of ALS pathogenesis, highlighting its role in disease progression.

Central to redox signaling within the redoxome, cysteine residues and post-translational modifications (PTMs) on cysteine sensitive proteins, referred to as “cysteinet” in the redox proteome, act as molecular switches that finely modulate protein activity. Their function is interconnected with reactive species to maintain and control cellular homeostasis in response to changes in the redox environment.

Interestingly, evidence points to the dual role of cysteine PTMs in neurodegeneration, particularly in ALS. Among the different modifications, reversible PTMs, such as S-nitrosylation may serve as protective mechanism against irreversible oxidative damage. Conversely, OS-induced modifications, such as disulfide bond formation, can impair function leading to protein misfolding and aggregation.

Understanding the interplay between redox dysregulation, cysteine sensitive proteins, and reactive species in NDDs is fundamental for elucidating disease mechanisms and identifying therapeutic targets.

In this perspective, this review aims to delve deeper into the redox code driven by oxidative cysteine PTMs, exploring their impact on neurodegeneration at the protein level, and highlighting the role of cysteine-driven redox signaling. Particular emphasis will be placed on the relationship between redox dysregulation mediated by Cys-modified proteins and ALS, highlighting this pathology as an example of a neurodegenerative disease wherein redox dysregulation is a distinct hallmark.

Exploring these processes may provide insights into potential therapeutic strategies to restore redox homeostasis and mitigate neuronal dysfunction and degeneration.

## 2. Redoxome and Cysteine Sensitive Proteins

Proteins, essential for cellular homeostasis and biological functions, are highly susceptible to damage from processes like oxidation.

PTMs are chemical alterations of a polypeptide chain occurring after translation, during the late stages of protein biosynthesis. There are more than 700 known PTMs, ranging from the enzymatic cleavage of peptide bonds to the covalent additions of chemical groups, lipids, carbohydrates, or even entire proteins [1]. These modifications can be reversible or irreversible, and commonly occur on 15 of the 20 proteinogenic amino acids side chains [2], particularly on serine, lysine, and cysteine [3].

PTMs enhance proteome complexity and diversity by altering physical and chemical properties of polypeptide chains, such as charge, hydrophobicity, and flexibility. Hence, they regulate protein folding, conformational stability, localization, and function playing pivotal roles in both physiological and pathological conditions, including neurodegeneration [4].

Within the redoxome, which comprises redox-regulated proteins [5], the importance of cysteine residues and cysteine-PTMs has garnered increasing recognition over the years [6]. Indeed, the term “cysteinet” has been meticulously coined to precisely delineate the subset of the redox proteome associated with cysteine residues, including all peptides and proteins containing functional redox cysteine residues in their structure [7]. This concept emphasizes the central role played by cysteine in modulating the cellular redox environment.

Although cysteine is not considered an essential amino acid, since it derives from the degradation of methionine, cysteine residues constitute approximately 2% of the total amino acid content in proteins [6]. Cysteine sulfur atoms can adopt a variety of oxidation states, ranging from −1 to +4, depending on pH and in response to numerous alterations in the cell’s redox state.

The thiol (–SH) side chains of cysteine are crucial for redox-mediated processes because of their unique chemical properties. These side chains can interact with metals, form disulfide bonds, and engage in redox reactions with ROS, RNS, and RSS [8].

Therefore, due to its high reactivity, cysteine has a wide spectrum of biological functions. For instance, its antioxidant properties, attributed to the easily oxidized thiol group, enable effective glutathione synthesis. Furthermore, under physiological conditions, the thiol group can be deprotonated to produce thiolate, which can bind to divalent metals such as zinc, iron, and cobalt, serving as active sites of enzymes. Additionally, the ability of two free cysteine residues to form disulfide bonds is crucial for maintaining protein stability. As a result, cysteine residues are primary targets of redox modification, influencing the activity of target proteins and determining susceptibility to oxidative damage and neurodegeneration. Specifically, cysteine sensitive proteins are involved in redox reactions, ranging from modulating catalytic functions of mitochondrial enzymes to acting as antioxidants. Redox modifications on cysteine regulate the thiol status of critical proteins involved in cellular redox balance, particularly in the central nervous system (CNS).

### Free Radicals in the Redox Balance Underlying Neurodegeneration

Free radicals, including ROS, RNS, and RSS, are ubiquitous in cells at basal levels and play crucial roles in various signal transduction processes [9].

Cellular free radicals are predominantly by-products of aerobic metabolism, with mitochondria being the primary source of ROS under physiological conditions (1–5%). Mitochondria, essential for adenosine triphosphate (ATP) production through aerobic respiration via oxidative phosphorylation (OXPHOS), also serve as critical hubs for redox regulation [10].

In particular, mitochondrial antioxidant mechanisms, including superoxide dismutase (SOD1), play key roles in maintaining ROS levels. However, when ROS levels exceed a certain threshold, antioxidant defense systems are activated. On the other hand, if the body’s antioxidant defenses fail to counteract such increased free radical levels, a condition of OS, characterized by the imbalance between pro-oxidant and antioxidant systems, occurs. Over the last years, OS has been comprehensively defined as redox dysregulation, focusing on the actual disruption of redox signaling and control [11].

The brain, as one of the most metabolically active organs, consumes 20% of inhaled oxygen in the body. Additionally, antioxidant systems within the brain are less effective compared to those in peripheral organs. Both high metabolic activity and relatively inefficient antioxidant systems make the brain particularly vulnerable to OS [12].

Mitochondrial dysfunction can lead to ATP depletion, increased ROS levels, and release of pro-apoptotic factors, all of which affect synaptic transmission, plasticity, and neuronal growth in the brain. Anomalies in mitochondrial morphology and function are prevalent in NDDs, with oxidative species and mitochondrial dysfunction serving as well-established features of these disorders [13], including ALS [14].

Cysteine-sensitive proteins, integral to the cysteinet network, are intricately involved in cellular redox signaling. Their function is interconnected with reactive species to maintain and control cellular signaling, ensuring cellular redox homeostasis [6]. Under specific pathological conditions, cysteine-containing proteins undergo redox modifications, leading to post-translational changes that alter protein function and contribute to neurodegenerative processes. In addition, the role of cysteinet is particularly significant in aging and aging-related neurodegenerative diseases [7,15].

## 3. Free Radicals and Cys-Sensitive Proteins Crosstalk: Cys-PTMs

Free radicals affect several cellular components, including nucleic acids, lipids, carbohydrates, and, primarily, proteins. Cysteine residues are particularly susceptible to redox modifications, and various PTMs may occur depending on the type of radical that interacts with them.

**Figure 1:**
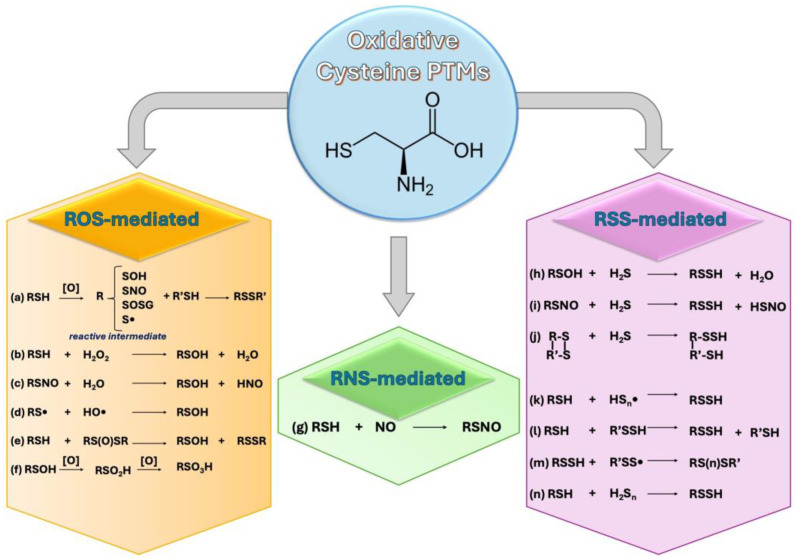
Schematic representation of oxidative cysteine reactions mediated by reactive oxygen (ROS), nitrogen (RNS), and sulfur (RSS) species. (**a**) S-thiolation; (**b**–**e**) sulfenylation; (**f**) sulfinylation and sulfonation; (**g**) S-nitrosylation; (**h**–**l**) persulfidation; (**m**) supersulfide formation; (**n**) polysulfide formation.

The following section provides an overview of cysteine PTMs, classified according to the reactive species (ROS, RNS, and RSS) responsible for inducing the modification (Figure 1).

Furthermore, key cysteine-modified proteins have been reported in Table 1, highlighting their impact on neurodegenerative processes at the protein level.

### 3.1. ROS and Cys-PTMs

In biological systems, the term ‘free radicals’ often refers to ROS, as most biologically relevant radicals are oxygen-centered. In cells, ROS include the hydroxyl radical (•OH), the superoxide radical anion (O_2_•^−^), and hydrogen peroxide (H_2_O_2_). ROS are produced through numerous cellular processes. Specifically, superoxide anions (O_2_•^−^) are predominantly generated by mitochondria and result from the one-electron reduction in molecular oxygen by enzymes in the mitochondrial respiratory chain during aerobic metabolism [12].

- S-Thiolation

S-thiolation is a reversible, non-enzymatic cysteine modification that forms mixed disulfides with low molecular weight (LMW) thiols in both physiological and pathological conditions. S-thiolated proteins rapidly form in response to OS, appearing within seconds of oxygen radical formation [41].

This modification can occur through thiol/disulfide exchange reactions or by the reaction of reactive intermediates, such as partially oxidized protein sulfhydryls (sulfenic acid or thiyl radical intermediates), with LMW thiol compounds, like glutathione, cysteine, and homocysteine (Figure 1a). S-thiolation can induce other PTMs such as S-cysteylation, S-homocysteylation, and particularly S-glutathiolation, which play an important role in regulating protein function and activity, as described below.

The reversible thiol-disulfide switch on cysteine residues regulates protein structure and function in response to redox changes [42,43]. Thiols, acting as antioxidant buffers, effectively scavenge reactive species to protect cells against oxidative injury. On the other hand, disulfide bonds can be reduced back to thiols, perpetuating redox equilibrium[42]. Thus, the balance between antioxidants and oxidants, reflected in the thiols/disulfides ratio, is crucial for assessing the organism’s dynamic redox system. Disruption of this switch can result in aberrant protein interactions, aggregation, dysregulated enzymatic activity, and compromised cellular signaling pathways, exacerbating neuronal damage and accelerating neurodegeneration [44].

- Disulfide formation

The dynamic switch between thiol and disulfide forms plays a key role in protecting thiol groups and preserving protein activity. This buffering system is finely tuned by the balance of redox couples such as cysteine/cystine and glutathione /glutathione disulfide (GSSG), as well as by redox-regulating proteins like thioredoxin (Trx), glutaredoxins (Grx), and peroxiredoxins (Prx).

The most well-known thiol oxidation reaction is the formation of disulfides (*R* (1)):

2*R*–SH → *R*–S–S–R + 2*e^−^* + 2H^+^ (1)

Disulfides bonds are reasonably stable intermediates and can undergo nucleophilic cleavage and thiol-disulfide exchange reactions. Disulfide exchange reactions are classified as S_N_2, in which a new disulfide and a new thiolate are formed by the thiolate attack toward a sulfur atom of a disulfide bond(*R* (2)) [41]:

*R*–S–S–*R* + *R′*–S^−^ → *R*–S–S–*R′* + *R*–S^−^ (2)

Many proteins, such as SOD1, require disulfide bond formation to attain the correct structure and become functional.

In SOD1, an intrasubunit disulfide bond between highly conserved pair of cysteines, namely Cys 57 and Cys 146, in the human form needs to attain the correctly folded quaternary structure and become enzymatically active. Indeed, when *SOD1* gene mutations occur, the lack of disulfide bond between Cys 57 and Cys 146 leads to protein misfolding and aggregation (Table 1), which are hallmarks of ALS pathogenesis [45], as discussed further on.

- Sulfenylation

Sulfenylation is considered the initial oxidation step of cysteinyl thiols, and the presence of cysteine sulfenic acid (Cys-SOH) has been recognized as a redox sensor in an increasing number of proteins [46]. Traditionally viewed as intermediates of other oxidation states, sulfenic acids are unstable, and highly reactive functional groups that can form through various reactions, as reported in Figure 1b–e. The high reactivity of -SOH is attributed to its dual electrophilic and nucleophilic nature.

The susceptibility of Cys residues to sulfenylation is mainly influenced by the nu-cleophilicity of the thiol, the surrounding microenvironment, and the proximity to ROS [47].

Sulfenic acids are involved in a wide variety of important chemical and biochemical reactions, including H_2_O_2_-mediated redox signaling. The antioxidant activity of many proteins, such as peroxiredoxins (Prxs), is attributed to the oxidation of cysteine to sulfenic acid during the catalytic cycle. However, under oxidative conditions, sulfenic acids can be further oxidized to form more stable products, namely sulfinic (Cys-SO_2_H) or sulfonic (Cys-SO_3_H) acids. This transition is an irreversible marker of oxidative damage [48]. For instance, both Prx2 and Prx3 show reduced activity when undergoing sulfenylation [49].

- Sulfinylation and Sulfonation

Cysteine sulfinylation (Cys-SO_2_H) occurs through the hyperoxidation of sulfenic acids by two-electron oxidants, such as peroxide species. At low levels of cellular ROS, sulfinylation can be reversed by the ATP-dependent reductase sulfiredoxin-1 [47]. However, in the presence of high cellular ROS levels, sulfynilation becomes irreversible, and cysteines can be further oxidized to sulfonic acids (Cys-SO_3_H) (Figure 1f) [50].

The irreversible oxidation of cysteine to sulfinic or sulfonic acid alters protein structure and function. Modifications with sulfinic and sulfonic acid increase with aging and can modify the activity of antioxidant enzymes such as Prx1 at Cys 51 [51]. Moreover, sulfinylation and sulfonation can lead to dysfunctions in the E3-ligase activity of parkin, resulting in abnormal ubiquitination and accumulation of toxic proteins (Table 1) [20].

### 3.2. RNS and Cys-PTMs

Historically, redox imbalance was primarily attributed to ROS; however, there is a growing awareness that RNS also significantly contribute to changes in redox homeostasis [52]. RNS include nitric oxide (NO•)-derived compounds. NO is produced by nitric oxide synthases (NOSs) from l-arginine and plays central roles in cell signaling through the regulation of several physiologic processes, such as vascular function, inflammation, and neuroplasticity [53]. Although NO• is a weakly oxidizing agent, it can react with O_2_•^−^ to produce peroxynitrite (ONOO^−^), leading to a decreased bioavailability of free NO and affecting most NO-mediated processes, including mitochondrial function [54].

In hydrophobic environment, NO can react with oxygen to produce nitrogen dioxide, which may trigger lipid peroxidation reactions and cell death [55].

- S-Nitrosylation

S-nitrosylation is a covalent PTM of cysteine residues, wherein NO group is attached to the -SH group of cysteine, forming an S-nitrosothiol (SNO) moiety. S-nitrosylation can occur through enzymatic and non-enzymatic mechanisms. Enzymatic S-nitrosylation is mediated by NOSs or by S-nitrosylases, which transfer the NO group from nitric oxide to cysteine residues in target proteins [56]. Non-enzymatic S-nitrosylation can occur via reaction between NO and thiol groups, under oxidative or nitrosative stress conditions [57]. The S-NO bond can also be formed via transnitrosylation mediated by NO-derived species, namely, LMW nitrosothiols (N_2_O_3_, e.g., S-Nitrosoglutathione (GSNO) and S-nytrosilated proteins [58] (Figure 1g).

Endogenous S-nitrosylated protein levels are usually low, and the SNO bond is highly labile and redox sensitive. The catalysis of this PTM occurs through multiple mechanisms, including thioredoxin, GSNO reductase, and trans-nitrosylation. S-nitrosylation induces a shift in protein structure that could alter protein–protein interactions, playing a crucial role in various physiological processes, including NO-mediated redox signal transduction and cell survival [59] and enabling further PTMs, such as acetylation and disulfide bond formation [60].

According to ROS levels, S-nitrosylation can be a reversible or irreversible modification [61]. At low ROS levels, S-nitrosylation not only scavenges NO to prevent it from reacting with ROS, but also protects cysteine thiols against ROS-mediated oxidation. However, when ROS levels increase, they can react with NO to form RNS, such as peroxynitrite (ONOO^−^) which induces oxidative damage, as observed in pathological conditions such as neurodegeneration [57]. Indeed, due to its radical nature, NO can exert both anti- and pro-oxidant effects depending on its concentration. For instance, exposure of Parkin to low levels of RNS increases its E3 ligase activity, while chronic exposition to RNS leads to the impairment of its ligase activity and repression of the transcriptional function of p53, contributing to the formation of misfolded or damaged proteins (Table 1) [31].

As reported in Table 1, dysregulation of S-nitrosylation pathways has been implicated in different neurodegenerative disorders. Protein disulfide isomerases can be inactivated through S-nitrosylation in ALS [32], as well as PRXs and DJ-1 (PARK7) (Cys 106) in PD [19,24]. Additionally, the mitochondrial fission protein Drp1 is S-nitrosylated (Cys 644) in postmortem brains and peripheral blood lymphocytes of AD patients [24].

### 3.3. RSS and Cys-PTMs

Recent advances in the field of sulfur biology and hydrogen sulfide (H_2_S) suggest RSSs, being signaling molecules along with ROS and RNS [62,63]. Mitochondria are rich sources of RSSs, actively generating sulfur compounds like glutathione persulfide (GSSH) during physiological activities, especially involving sulfide oxidation [64]. The most biologically important RSS include H_2_S and sulfane sulfur containing compounds, namely thiosulfate, inorganic and organic polysulfides, LMW persulfides, and protein persulfides [65].

H_2_S is a gas transmitter endogenously produced by cystathionine beta synthase (CBS) and cystathionine gamma lyase (CSE) in the trans-sulfuration pathway from homocysteine [65] by 3-mercaptopyruvate (3-MP) and sulfurtransferase (MST) from 3-MP in combination with cysteine aminotransferase (CAT) [66], and by D-amino acid oxidase (DAO), from D-cysteine [67]. H_2_S can modulate several features of cellular physiology and pathology, via S-sulfhydration (persulfidation) of cysteines in target proteins mainly in the CNS [68,69,70,71]. Thiol groups of cysteines are the main targets of redox changes, thus the identification of an increasing number of sulfur-derived radicals adds additional layers of complexity in the redox signaling processes.

- S-sulfhydration (persulfidation)

As Mustafa et al. stated, S-sulfhydration (or persulfidation) is an oxidative PTM through which H_2_S modulates several physiological processes in CNS [72]. This modifi-cation is characterized by the addition of sulfur atoms to cysteine residues in proteins, involving the conversion of a cysteine –SH group into a –SSH (persulfide), thereby regulating protein functions [73].

It can be achieved through non-enzymatic reaction of H_2_S with various cysteine modifications, such as sulfenic acids (–SOH) (Figure 1h), S-nitrosated cysteines (–SNO) (Figure 1i), and cysteine disulfides (–S–S) (Figure 1j), respectively. In addition, persulfides can be formed by the reactions between thiol groups of cysteine and sulfide radicals (Figure 1k), by the ‘trans-sulfhydration’ reactions (Figure 1l), or by reacting with LMW persulfides or hydropolysulfides (Figure 1m).

Similar to ROS and RNS, the effects of RSS on target proteins depend on their concentration. Indeed, H_2_S follows a bell-shaped model, wherein steady-state levels act within a narrow optimal nanomolar concentration range [74,75].

Within this concentration range, persulfidation acts as a rescue pathway, preventing cysteine hyperoxidation to sulfinic and sulfonic acids, and alleviating hyperoxidation-induced protein dysfunction. Protein persulfidation exerts anti-aging, anti-apoptotic and anti-inflammatory effects, playing a potential protective role against neurodegeneration [9,72]. For example, persulfidation assumes a protective role in PD by modulating the activity of SIRT1 to increase the autophagic process [40]. Moreover, persulfidation of GSK3β (Cys 218) inhibits tau phosphorylation in AD as reported in Table 1 [37].

Conversely, persulfidation of liquoral transthyretin (Cys 10) has been observed in patients with MS; however, it seems to correlate with a reduced protein function [22,39] (Table 1).

Other NDDs, such as ALS [76,77] and Down syndrome [78,79], are characterized by disruption of H_2_S homeostasis leading to cellular and systemic dysfunction although the role of persulfidation in these diseases needs to be clarified.

Recently it has also been introduced the term “supersulfide” which include an umbrella of sulfur species such as hydropersulfides (RSSHs) and polysulfide species (RSS_n_R; n > 1, R = hydrogen, and alkyl or cyclic sulfurs) [80] (Figure 1m). They can accept electrons produced by the electron transport chain (ETC), contributing to sulfur respiration, and they can suppress lipid oxidation and ferroptosis, thus exerting antioxidant effects [81]. However, their role needs to be clarified in the CNS.

- Polysulfides

Polysulfides (H_2_S_n_, n = 2–8) are H_2_S-derived endogenous molecules, with the sulfur atom in the oxidation states of 0 or −1, generated by the incomplete oxidation of H_2_S [82]. H_2_S_n_ can also be generated by CSE and CBS through the conversion of persulfides, such as CysSSH, GSSH, or by the oxidation of H_2_S [83,84].

H_2_S can be oxidated through reactions with ROS, mitochondrial SQR, cytosolic copper/zinc superoxide dismutase (Cu/ZnSOD), and manganese superoxide dismutase (MnSOD). Additionally, polysulfides can be produced by the oxidation of 3-MP by 3-MST and by H_2_S donors such as GYY4137 and NaHS. This modification can occur through various pathways, including enzymatic and non-enzymatic processes, as well as by the biochemical interaction of H_2_S and NO [85,86], and the decomposition of –SSNO [87].

Similarly to H_2_S, polysulfides can act as signaling molecules and induce S-persulfidation by the addition of multiple sulfur atoms to cysteine residues (Figure 1n). The biological significance of polysulfide modifications is diverse. They can alter protein structure, stability, and function affecting various cellular processes. In PD, polysulfides play a protective role by activating Parkin through sulfhydration of cysteines (Cys 59, Cys 95, Cys 182, Cys 212 and Cys 377) leading to degradation of misfolded proteins and supporting neuroprotection [38].

### 3.4. Other Cys-PTMs

Due to the nucleophilicity and redox sensitivity of the thiol side chain, cysteine residues are susceptible to various PTMs. In addition to the mentioned modifications induced by reactive species, oxidation PTMs also include those induced by glutathione. Along with the oxidative PTMS, two other categories should also be considered. They include lipid PTMs involving cysteine lipidation, such as S-palmitoylation and S-prenylation, and molecules PTMs encompassing a range of modifications caused by reactive metabolites, including S-carbonylation [88,89].

Although these modifications are not the primary focus of this review, some details are provided due to significant close association with many diseases, particularly neurodegenerative disorders.

#### 3.4.1. Ox-Cys-PTMs

- S-glutathionylation

Among the various types of S-thiolation, S-glutathionylation emerges as the most significant PTM. S-glutathionylation is a reversible oxidative modification in which GSH is conjugated to an exposed cysteine residue. The occurrence of this PTM is linked to the cellular redox state, primarily relying on the reduced to oxidized glutathione (GSH/GSSG) ratio [90]. It acts as a protective mechanism against the irreversible oxidation of thiols [91]. S-glutathionylation can occur both enzymatically or non-enzymatically, through the interaction of thiyl radicals, sulfenic acids, or protein S-nitrosothiols with GSH, or through thiol/disulfide exchange, while its reversal can be mediated by enzymes like GRX or GST [90]. S-glutathionylation has notable effects on mitochondrial bioenergetics by modulating OXPHOS, through the modification of mitochondrial complexes [92], as well as tricarboxylic acid (TCA), inhibiting α-ketoglutarate dehydrogenase complex (KGDH) [93] and aconitase [94]. Moreover, this PTM is implicated in the inhibition of long-chain fatty acid oxidation, decreasing the capacity of the carnitine antiporter CACT [95]. Additionally, S-glutathionylation can influence mitochondrial morphology by modulating proteins such as mitochondrial dynamin like 120 kDa protein (OPA1) and Mitofusin 2 (MFN2) [96]. Given its multifaceted role in mitochondrial dynamics, it is not surprising that S-glutathionylation is implicated in neurodegenerative diseases [97,98].

For instance, elevated levels of glutathionylated proteins have been shown in the inferior parietal lobule of postmortem brains of patients with AD [99]. Key proteins like Tau [100] and mitochondrial human branched-chain aminotransferase protein (hBCAT) [101] have been identified to undergo this modification. In PD, glutathionylation can affect the activity of parkin [102] and DJ1 [103]. In ALS, following glutathionylation on Cys 111, SOD1 can dissociate and aggregate independently of the presence of the Cu/Zn center and the disulfide bond formation [104]. Moreover, S-glutathionylation contributes to ALS progression by inhibiting of PDI1 activity, thereby enhancing mutant SOD1 aggregation [105].

Furthermore, S-glutathiolation can be induced by RNS. Peroxynitrite ONOO^-^ reacts with GSH, inducing S-glutathionylation on the pro-peptide of MMP2 cysteine residue, which leads to matrix remodeling, involved in neurodegeneration [106,107].

- S-Carbonylation

S-carbonylation involves the covalent attachment of carbonyl groups (such as aldehydes and ketones) to the thiol group of cysteine residues.

This modification typically results from oxidative stress, where reactive carbonyl species, such as those formed from lipid peroxidation (e.g., 4-hydroxynonenal), react with cysteine thiols [108]. Carbonylated proteins often exhibit impaired function, disrupting cellular homeostasis, particularly in neurons where precise protein function is critical.

Due to OS, increased levels of carbonylated proteins have been associated with the pathological mechanisms of several diseases, including AD [109], PD [110], and MS [111], contributing to protein aggregation, and neuronal death.

#### 3.4.2. Lipid Modifications

- S-Acylation

S-acylation is a reversible PTM wherein medium-chain or long-chain fatty acids attach specific cysteine residues via a labile thioester linkage [112]. This process commonly involves the addition of the 18-carbon chain of stearic acid, or the 16-carbon chain of palmitic acid. In the latter case, the modification is known as S-palmitoylation. S-acylation is mediated by a family of S-acyltransferases that contain a conserved Asp-His-His-Cys (DHHC) sequence and a zinc finger active site (ZDHHC) [112]. Conversely, it can be reversed by various acyl-thioesterases, containing a conserved catalytic serine. Acting as a dynamic on/off switch, S-acylation regulates changes in protein localization, leading to association with specific membrane domains, as well as structural alterations. This PTM governs many cellular processes, including signaling, inter-organelle and inter-cell communication, energy metabolism, and tissue function. Particularly, it is involved in synaptic communication, neuronal plasticity, and autophagy. Consequently, dysregulations in S-acylation and deacylation dynamics are implicated in the pathogenesis of neurodegenerative disorders [112,113].

- S-palmitoylation

S-palmitoylation, the most prevalent form of protein acylation, involves the conjugation of palmitate to the thiol group of cysteines via a labile thioester bond. This PTM is mediated by palmitoyl acyl-transferases with ZDHHC active site domains. On the other hand, depalmitoylating enzymes, such as acyl protein thioesterases, protein palmitoyl thioesterases, and α/β-hydrolase domain proteins, can reverse it [114,115]. S-palmitoylation leads to changes in protein localization, anchoring them to membranes. Consequently, the switch between the palmitoylated and depamitoylated form redirects proteins to and from membranes based on local signaling factors [116]. In mammals, S-palmitoylation affects more than 50% of synaptic proteins, and plays a central role in protein shuttling between intracellular compartments [117]. This PTM regulates neuronal protein trafficking and function, as well as proteostasis, by determining protein localization involved in the pathogenesis of numerous NDDs [118].

In Huntington’s disease (HD) a decrease in palmitoylation of mutant Huntingtin at Cys 214 occurs, resulting in the formation of insoluble protein aggregates [119]. Many key proteins involved in AD pathogenesis are found to be palmitoylated, including amyloid precursor protein (Cys 186, Cys 187) [120], APH1aL (Cys 182, Cys 245), Nicastrin (Cys 68, Cys 69) [121], and the Fyn kinase, (Cys 3, Cys 6) [122]. SOD1 has also been found to be palmitoylated at Cys 6 in ALS [123].

- S-Prenylation

S-prenylation, also known as isoprenylation, is an irreversible PTM that occurs shortly after translation. It involves the covalent attachment of a prenyl group, such as farnesyl or geranylgeranyl pyrophosphate, intermediates of the cholesterol metabolism, to cysteine residues. Prenylation is catalyzed by protein prenyltransferases, including farnesyltransferase and geranylgeranyl transferase I and II and III [124].

Target proteins typically feature a CAAX prenylation motif, which contains a cysteine and two aliphatic amino acids. Proteins with a leucine C-terminal amino acid usually undergo geranyl-geranylation, while those with a serine, methionine, ala-nine, or glutamine terminal amino acid are typically farnesylated [125]. The covalent addition of these isoprenoids to cysteine residues mediates changes in protein localization, targeting them to membranes, and influencing protein function and protein–protein interactions. Several processes, including signal transduction, proliferation, apoptosis, vesicular transport, cytoskeleton organization, and cell motility, depend on the participation of prenylated proteins. Protein prenylation occurs ubiquitously, and defects in isoprenoid biosynthesis or regulation are implicated in aging and in the pathogenesis of various diseases, from cancer to metabolic and neurodegenerative diseases [126]. Particularly, defects in isoprenoid regulation and protein prenylation are associated with AD [127].

Indeed, abnormal levels of farnesyl or geranylgeranyl pyrophosphate have been observed in the brain of AD mice and patients [128], and these isoprenoids enhance amyloid beta production [129]. In addition, prenylation seems to play a role in ALS progression, enhancing autophagy in vitro through the modulation of RABGGTB [130]. Conversely, farnesylation appears to have a protective role in PD, by decreasing the degeneration of dopaminergic neurons, through the inhibition of the parkin-interacting substrate PARIS [131].

#### 3.4.3. Molecules PTMS

- S-homocysteinylation

S-homocysteinylation represents another form of S-thiolation, characterized by the formation of a disulfide bond between homocysteine (Hcy) and Cys residues [132].

Hcy is a common intermediate of one-carbon metabolism, derived from the essential amino acid methionine. Although not proteinogenic, homocysteine plays a crucial role in the synthesis of sulfur-containing amino acids such as methionine, cysteine, and homocysteinylated proteins. S-homocysteinylation occurs through non-enzymatic processes [133], either by the direct addition of reduced Hcy to available cysteinyl residues or through disulfide exchange reactions with other S-homocysteinylated proteins, such as transthyretin [134]. Homocysteine, along with the process of homocysteinylation, is im-plicated in the pathogenesis of NDDs, due to its involvement in facilitating the aggregation of misfolded proteins. Hyperhomocysteinemia is considered a risk factor for AD and dementia, as it can induce vascular dysfunction promoting the formation of fibrin clots [135]. Additionally, homocysteinylation promotes the interaction between fibrinogen and Aβ-peptide, facilitating its deposition in the cerebral vessels [136,137]. Furthermore, S-homocysteinylation of Prx1 at its catalytic Cys 52, may inhibit its peroxidase and chaperone activities, resulting in decrease in the antioxidant response [138].

- Non-canonical ubiquitination

Ubiquitination is a PTM that commonly targets lysine residues of proteins, leading to protein degradation by adding ubiquitin molecules. In rare cases, cysteine ubiquitination, also known as non-canonical ubiquitination, may occur [139,140]. This PTM involves the formation of a thioester bond between the Gly 76 of ubiquitin and a cysteine residue of the target proteins. The resulting adducts are thermodynamically less stable than those generated on lysine residues. However, due to the soft nucleophilic nature of the sulfur lone pair, this reaction occurs relatively quickly. The rapidity of this reaction is particularly important in signaling events requiring a swift response.

Ubiquitination plays a key role in the unfolded protein response system and autophagy. Alterations in these cellular processes may contribute to the pathogenesis and progression of NDDs [141].

## 4. Redox Dysregulation in Amyotrophic Lateral Sclerosis

Amyotrophic lateral sclerosis (ALS) is a neurodegenerative and devastating disorder characterized by the death of motor neurons in spinal cord, brain stem, and motor cortex, with still no convincing therapy. Several etiologic factors can contribute to such neurodegeneration, including genetic and environmental ones. While the majority are sporadic (sALS), approximately 5–10% of ALS cases are familial (fALS). Several genes have been implicated in familial ALS, including *SOD1* (superoxide dismutase 1), *C9orf72* (chromosome 9 open reading frame 72), *TARDBP* (TAR DNA-binding protein), *FUS* (fused in sarcoma), and others. Mutations in these genes disrupt cellular functions such as protein quality control, RNA metabolism, and mitochondrial dynamics, leading to motor neuron dysfunction and degeneration [142].

Despite the underlying causes of motor neuron death remain largely unknown, a scenario of strong OS and mitochondrial dysfunction has long been associated with ALS [14,143,144]. Evidence points out that this dysregulation could be a significant driver of neurodegeneration worthy of greater importance than previously recognized. Regulation of thiol redox balance is critically important in maintaining the redox balance for multiple metabolic and signaling processes. Central redox regulatory mechanisms, including thioredoxin (Trx) and GSH systems, and related redox-sensitive proteins, are perturbed in ALS [145].

In this context, impaired antioxidant defenses lead to an increase in reactive species, damaging cellular components such as proteins, lipids, and DNA, ultimately resulting into neuronal dysfunction and death.

Furthermore, RSS interacting with cysteine sensitive proteins can also modulate redox signaling in ALS [76].

**Figure 2:**
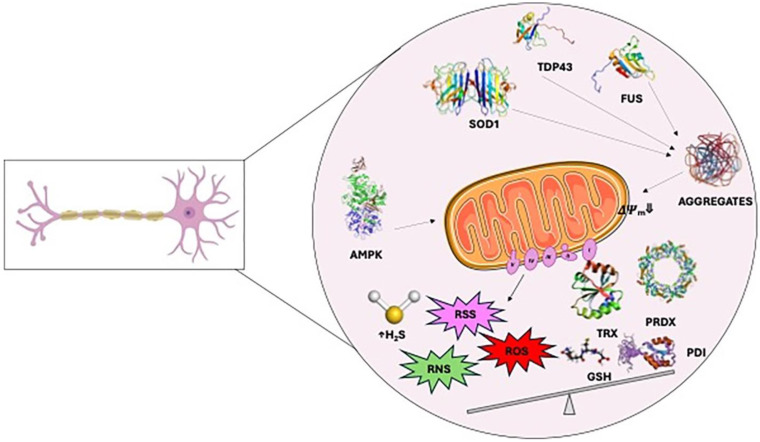
Schematic representation of cysteine modified proteins involved in the pathogenesis of amyotrophic lateral sclerosis, underlying motor neuron death.

Dysregulation of the redox state of cysteines, related to PTMs, seems to be involved in several mechanisms that are important for the maintenance of correct protein folding and a function in ALS [146] (Figure 2).

### 4.1. Cysteine Modifications in ALS

Cysteine residues and related PTMs assume a prominent role in ALS pathogenesis.

Firstly, cysteine residues are essential for maintaining cellular redox balance through two primary disulfide reductase systems, the Trx and GSH systems. The cysteine residues within thioredoxin and tripeptide glutathione (GSH, g-L-Glutamyl-L-cysteinylglycine) sequences participate in a complex network of redox reactions [147].

Secondly, cysteines are fundamental in the formation of protein inclusions or aggregates observed in ALS, which are common features in both sporadic and familial forms of the disease. Aberrant cysteine modifications, resulting in proteostasis loss, are found in many proteins involved in ALS pathogenesis, including SOD1, TDP43, peroxiredoxins (PRXs), protein disulfide isomerases (PDIs), AMP kinase, and fibroblast growth factor 2 (FGF2). Additionally, redox-sensitive cysteine thiols play critical role in cellular processes including redox signal transduction (e.g., Nrf-2, NF-kB) [148]. Enzyme activity in various pathways and the function of transcription factors acting as redox sensors depend on disulfide bond formation to maintain redox homeostasis [148]. Hereby, key cysteine modified proteins involved in these pathways are described and represented in Figure 2.

#### 4.1.1. Cysteine Sensitive Proteins and Redox Unbalance

The whole redox state of the cell is regulated by two main cellular disulfide reductase systems: Trx and GSH systems.

Alongside these systems, redox networks include antioxidant enzymes such as SOD, and catalase (CAT), non-enzymatic molecules like peroxiredoxins, and essential dietary factors, such as vitamin C, vitamin E, and carotenoids.

Defective cellular redox conditions can result in increased levels of reactive species, leading to irreversible thiol modifications such as sulfinic (–SO_2_H) or sulfonic acid (–SO_3_H) formation. These conditions can induce protein misfolding and anomalous protein aggregation, as observed in ALS.

- In addition, the dysregulation of iron metabolism could play a role in the redox unbalance of ALS, leading to iron accumulation in both sporadic and familial forms of ALS, including mouse models [149,150].Thioredoxin (Trx) system

The thioredoxin (Trx) system plays a crucial role in regulating redox balance and cellular functions in response to redox signals and stress, which are implicated in cell survival in various conditions, including neurodegenerative diseases [151].

The Trx system consists of Trx, thioredoxin reductase (TrxR), thioredoxin peroxidase (TrxP), and nicotinamide adenine dinucleotide phosphate (NADPH).

Trx is a small 12 kD protein with a conserved sequence, Cys-Pro-Gly-Cys, essential for its function as an electron donor of peroxiredoxins [152].

TrxR catalyzes the reduction in disulfide bonds using NADPH to convert the oxidized form of Trx (Trx-(S)_2_) to its reduced form (Trx-(SH)_2_), while TrxP reduces peroxides and restores Trx to its active form [153].

Trx and TrxR are widely expressed in various tissues including the brain, under-scoring their importance in cellular physiology and maintenance.

Dysregulation of the Trx system has been implicated in ALS pathogenesis. Specifically, Trx protein expression is upregulated in postmortem spinal cords of ALS patients and in erythrocytes of fALS with mutant SOD1 suggesting a potential compensatory response to increased OS in ALS pathology [154,155].

- Glutathione system

The glutathione system includes glutathione reductase (GR), NADPH, GSH, and glutathione peroxidase (GPx). GR is involved in converting glutathione disulfide (GSSG) to GSH, whereas GPx is involved in the reverse reaction [145].

Dysregulation of the GSH system has long been recognized in ALS. Decreased GSH levels have been shown in the motor cortex, serum, and fibroblasts of human sALS patients [156,157], as well as in SOD1^G93A^ mice models [158].

- Peroxiredoxins

Peroxiredoxins (Prxs) are a family of thiol-dependent peroxidases involved in catalyzing reactive species [159]. In humans these enzymes are classified into six subfamilies (Prx1 to Prx6) based on sequence and structural homology.

Prxs do not require special cofactors for their activity and catalyze a two-step reaction, known as Prx cycle, which revolves around a redox-active cysteine called peroxidatic cysteine (CP). In the first step of this cycle, the peroxidatic cysteine thiolate (RS^−^) is oxidized by peroxide to form sulfenic acid (R-SOH) or sulfenate (R-SO^−^). The second step consists of the regeneration of the peroxidatic cysteine. This step is performed through the reductive recycling by other cysteine-sensitive proteins, called resolving, such as Trx or Trx-like proteins, which attack the R-SOH to form a water molecule and a disulfide bond in the protein. The catalytic cycle concludes with the reduction in the disulfide bond through the afore mentioned reducing agents [160].

Prxs play a dominant role in the antioxidant response by regulating peroxide and peroxynitrite levels in mammalian cells, and assume a prominent role in neuro-degenerative processes, characterized by a notable redox dysregulation [159,161] such as in ALS [162,163]. However, it is not yet clear how these proteins contribute to ALS pathology. Presumably, the irreversible oxidation of the catalytic cysteine to sulfinic and sulfonic acid plays a key role [51]. Indeed, PRXs with hyperoxidized cysteines exhibit decreased activity, exacerbating redox imbalance [164]. Prx3, found in mitochondria, is downregulated in SOD1^G93A^ transgenic mice and in spinal cords of sALS and mutant SOD1 ALS patients, highlighting a loss of redox regulation and antioxidant defense mechanisms in ALS [163]. Conversely, Prx6 was found to be upregulated in spinal motor neurons of SOD1^G93A^ mice[165], as well as Prx 1 was upregulated in NSC-34 cells expressing SOD1^G93A^ [166].

- Hepcidin

Iron dysregulation is a significant feature in ALS, with substantial evidence indicating its role in promoting OS and damage to motor neurons. Hepcidin, a peptide primarily synthesized in the liver, plays a critical role in systemic iron regulation by inhibiting ferroportin, leading to its internalization and degradation. Hepcidin contains eight cysteine residues that form four disulfide (S-S) bonds, which are essential for its structural integrity and function [150]. As far as we know, hepcidin, along with ferritin and transferrin, are accepted markers of inflammation and iron metabolism, respectively [167]. However, there is currently no direct evidence supporting the hypothesis of Cys-PTMs related to hepcidin. Nonetheless, it is plausible that reactive species could modify the structure of hepcidin, potentially leading to the loss of its functional conformation and impairing its ability to regulate ferroportin, thereby disrupting iron homeostasis. Further research could provide deeper insights into the interaction between hepcidin and ferroptosis in ALS mechanisms.

#### 4.1.2. Cysteine Sensitive Proteins and Protein Aggregation

Accessible cysteine residues are key elements for the formation of protein inclusions and aggregates. Anomalous protein aggregates and misfolded proteins are common hallmark in ALS, observed in both sporadic genetic forms. Several proteins are known to aggregate, including SOD1, TDP-43, FUS, vasolin-containing protein (VCP), ubiquilin-2 and dipeptide repeats produced by unconventional RNA-translation of the GGGGCC expansion in *C9orf72*.

- SOD1

Human *SOD1* gene maps on the 21q22 chromosome and encodes the main antioxidant enzyme of the eukaryote cell, SOD1.

SOD1 is a cytosolic homodimeric enzyme, with each monomer composed of 153 amino acids, forming eight β-antiparallel strands, and containing four cysteines at positions 6, 57, 111 and 146 respectively. Proper functioning of SOD1 requires PTMs, specifically intramolecular disulfide bond formation between Cys 57 and Cys 146 for correct folding, binding of zinc and copper metal ions, and exposure of a hydrophobic region crucial for organizing SOD1 dimers [168]. In mitochondria, SOD1 switches from its apoenzyme state to its mature form through the establishment of disulfide bonds and insertion of copper ions by the copper chaperone for superoxide dismutase (CCS) [169]. Conversely, unfolded SOD1 translocates from the cytosol into mitochondria, through the outer mitochondrial membrane (OMM) via the translocator TOM [168]. This maturation process ensures that SOD1 remains within mitochondria and does not leak back into the cytosol.

The intracellular localization of SOD1 is closely linked to CCS, which acts as a redox sensor determining in turn SOD1’s subcellular localization. At high oxygen levels, CCS remains in the cytosol to fold apoenzyme SOD1. However, in hypoxic conditions, CCS translocates to the mitochondrial intermembrane space (IMS), facilitating the formation of sulfur bound and metal copper incorporation to form mature SOD1 in preparation for a burst of free radical production [170].

Furthermore, changes in redox potential can reduce the disulfide bond, exposing free cysteines at positions 6 and 111. These cysteines can react with thiol groups of other molecules, leading to aggregate formation. Cys 111 is particularly prone to alkylation through intermolecular bonds. Its sulfhydryl group is highly sensitive to redox changes, undergoing further oxidation via sulfinylation and sulfonation. Modified Cys 111 promotes aggregation independently of the disulfide bond status, disrupting redox homeostasis and increasing reactive species production [104].

Aggregation of misfolded SOD1 alters redox homeostasis, leading to the increase in reactive species [171], and amplifies mitochondrial dysfunction due to the aggregates’ presence in the mitochondrial intermembrane space [172]. Accumulation in mitochondrial SOD1, together with mitochondrial dysfunction, are key features leading to the motor neuron death [172].

Likewise, the accumulation of SOD1 in mitochondria has been proposed as a particular hallmark of motor neuron degeneration. In fact, signs of mutant SOD1 accumulation and, consequently evident mitochondrial dysfunction have been demonstrated in the spinal cord [173] and neurons [174].

Moreover, SOD1 accumulation increases with disease progression [173].

The thiol group of Cys 111 can also be modified through S-glutathionylation, re-sulting in monomer formation and aggregation [175].

SOD1 can also undergo S-palmitoylation on Cys 6. This modification is increased in ALS and mainly takes place on reduced disulfides, suggesting that monomeric SOD1, generated by the presence of mutations to SOD1 gene and/or redox dysregulation, is the primarily targeted species [123].

- TDP43

Mutations in the *TARDBP* gene, which encodes TDP43 protein, are implicated in ALS pathogenesis.

TDP43 is a highly conserved and ubiquitously expressed RNA/DNA-binding protein involved in various steps of RNA biogenesis and processing, including the splicing regulation of many non-coding and protein-coding RNAs, critical for neuronal survival [176,177].

In physiological condition, TDP-43 is mainly found in the nucleus; however, pathological inclusions containing TDP-43 are predominantly found in the cytosol.

In fact, a prominent hallmark of ALS is the loss of functional TDP-43 in the nucleus and its increased deposition into cytoplasmic inclusion bodies in the brain and spinal cord. The majority of sporadic ALS patients exhibit TDP-43 protein deposition in neuronal inclusions, highlighting its pivotal role in ALS [178]. Therefore, understanding the molecular mechanisms underlying TDP-43 pathology is crucial for advancing ALS therapies. The pathological features of TDP-43 proteinopathies include nucleus-to-cytoplasmic mislocalization, deposition of ubiquitinated and hyper-phosphorylated TDP-43 into inclusion bodies, protein truncation leading to the formation of toxic C-terminal TDP-43 fragments and protein aggregation. Sporadic or familial mutations can exacerbate these detrimental effects, potentially causing early disease onset [176]. Proteolytic cleavage and consequent formation of C-terminal TDP-43 fragments (CTFs) of approximately 35 kDa and 25 kDa are one of the most common TDP-43 species observed in ALS and FTD brain inclusions [179].

TDP-43 can be subject to different PTMs including cysteine oxidation, but also phosphorylation, ubiquitination, acetylation, and sumoylation [176].

The sequence of TDP43 contains six cysteine residues: Cys 39 and Cys 50 in the N-terminal domain; Cys 173, Cys 175, Cys 198, and Cys2 44 located in the two RNA recognition motifs (RRMs). Among these, the latter four are the primary redox-regulated cysteines, with Cys 173 being preferentially oxidized due to its propensity to react with Cys 175, forming intramolecular bonds. Under OS conditions, these residues undergo oxidation and disulfide bond formation, leading to the generation of cross-linked species prone to aggregation [18].

Full-length TDP43, regardless of ALS-linked mutations, relocates from the nucleus to the cytosol, forming oligomers [18,180]. TDP43 cross-linking alters the protein’s conformation, impairs its nuclear function, and reduces its solubility.

Notably, the 35 kDa and 25 kDa isoforms resulting from proteolytic cleavage of full-length TDP43, which are found in the insoluble fraction in patients are pre-dominantly included in cysteine-dependent oligomers [180]. Overall, given that aggregates formed by SOD1 and TDP43 are present in all ALS patients (including those with sALS), these findings underscore the pivotal role of cysteine redox state in ALS pathogenesis.

- FUS

Fused in sarcoma (FUS), encoded by chromosome 16, is a widely expressed protein in the nucleus of most human tissues and acts in several cellular processes including DNA repair and RNA processing specifically in the brain. Additionally, FUS supports synaptic plasticity by regulating mRNA transport [181].

FUS protein consists of a sequence of 526 amino acids including repeated argi-nine/glycine-rich (RGG) domain, an RNA/DNA-recognition motif (RRM), a zinc-finger (ZnF) motif, and a nuclear localization signal (NLS). In the spinal cord of ALS patients with FUS mutations, FUS aggregates exhibit abnormal cytoplasmic localization, highlighting potential alteration in the nucleocytoplasmic transport [182].

Certain ALS-associated FUS mutants promote the abnormal cytoplasmic accumulation of FUS inclusions [183].

Several PTMs, including hypermethylation and phosphorylation [184], lead to anomalous cytoplasmic localization and aggregation of FUS [185]. However, glutathionylation seems the major redox-sensitive PTM that can alter FUS activity and stability [186].

In the brains of the Drosophila model, glutathionylation of FUS at Cys 447 in the RanBP2-type ZnF domain induces FUS aggregation. Conversely, overexpression of glutathione transferase omega 2 (GstO2) reduces cytoplasmic FUS aggregates by deglutathionylation, preventing neurodegeneration and mitochondrial dysfunction. Thus, cysteine glutathionylation of FUS might be a promising therapeutic strategy for FUS-associated neurodegenerative diseases [186].

#### 4.1.3. Cysteine Sensitive Proteins and Redox Signaling

As previously discussed, the dysregulation of the redox state of cysteines seems to play a significant role in several mechanisms crucial for maintaining proper protein folding and activity in ALS.

Additionally, other aspects of cysteine metabolism may also be relevant in the pathogenesis of ALS, including the direct oxidation of cysteines in proteins that are essential for motor neuron metabolism and survival.

- Protein Disulfide Isomerases

Protein disulfide isomerases (PDIs) are members of the Trx family, located in the endoplasmic reticulum (ER), which play a critical role in regulating intracellular redox processes.

The involvement of PDIs has been highlighted in ALS pathogenesis. PDI levels are upregulated in transgenic ALS models and in spinal cord tissues of ALS patients [145,187].

PDIs exhibit chaperone activity ensuring correct protein folding and preventing protein misfolding and aggregation. Moreover, PDIs show oxidoreductase activity through the formation/breakage of disulfide bonds mediated by cysteine residues within their active site [187].

Mutations in PDI, such as D292N and R300H, that occur in ALS result in the loss of oxidoreductase activity and are considered as genetic risk factors for ALS, underscoring the importance of PDI redox activity in such disease [188].

Furthermore, PDIs have been shown to be protective against ALS phenotype, rescuing motor dysfunction and axonopathy in ALS zebrafish models with mutant SOD1 [188]. In addition, PDIs are also protective against mutant FUS, restoring alterations in nuclear import, preventing the formation of mutant FUS aggregates in neuronal cell line and primary cultures [188,189].

Cysteine residues of PDI family members can be modified by PTMs, such as disulfide formation [146], and S-nitrosylation [190,191]. S-nitrosylation in the active site of PDIs inhibits their enzymatic activity, contributing to the formation of misfolded protein inclusions, such as SOD1 [32] and FUS aggregates [192] in ALS.

Aberrant upregulation of S-nitrosylated PDIs has been detected in post-mortem spinal cords of ALS patients, compared to controls [193]. Conversely, a decrease in S-nitrosylase activity of S-nitrosoglutathione reductase has been observed in neuronal cells of fALS patients with SOD1 mutation [194].

- AMPK

Adenosine 5′-monophosphate (AMP)-activated protein kinase (AMPK) is a key sensor and a metabolic regulator that plays a central role in maintaining cellular energy homeostasis, which is particularly relevant in neurodegenerative diseases, as reviewed by [195]. It is activated in response to changes in AMP-to-ATP ratio, reflecting low cellular energy status.

While specific cysteine modifications on AMPK are less extensively characterized compared to other PTMs, like phosphorylation or acetylation, cysteine modifications can play significant roles in regulating protein activity and redox signaling.

AMPK activity itself can be regulated by the cellular redox state [196,197]. AMPK has various redox sensitive cysteines and can either be activated or inhibited by their modifications. For example, cysteine residues such as Cys 299 and 304 can be oxidized and S-glutathionylated by H_2_O_2_, leading to the formation of disulfide bonds and the activation of the enzyme [197]. Conversely, oxidation of Cys 130 and Cys 174 residues of the α-subunit can inhibit AMPK, promoting its aggregation and disrupting its interaction with upstream kinases [198].

AMPK seems to have a role in the pathogenesis of ALS wherein an increased activity is shown in spinal cord cultures expressing mSOD1, as well as in the spinal cord from mSOD1 mice and patients [199].

AMPK activation is also associated with TDP43 mislocalization in motor neurons and energy dysfunction [200].

Additionally, AMPK can be modulated by the mitochondrial enzyme CARS that is involved in RSS mediated signaling [201].

The relationship between SIRT1 and AMPK is noteworthy in relation to ALS [202].

Sirtuins (SIRTs) members are NAD^+^ dependent histone deacetylases, playing a regulatory role in apoptosis, metabolism and aging. In mammals, the SIRT family contains seven members, SIRT1–7. Among the different members, SIRT1 is widely distributed in human tissues and organs, especially in the nervous system. SIRT1 signaling participates in autophagy, mitochondrial biogenesis, and cell survival processes, suggesting its potential role in ameliorating neurodegenerative diseases [203].

AMPK can activate SIRT1 by promoting the formation of NAD^+^ (oxidized). This activation, in turn, is promoted by sestrin2, an antioxidant cysteine sulfoxide reductase, which acts via the mTOR (mammalian target of rapamycin) and Nrf2 signaling pathways.

The Sestrin2/AMPK/SIRT1 axis has a strong impact on maintaining cellular metabolism, and neuroprotection by decreasing oxidative stress and mitochondrial dysfunction.

The upregulation of AMPK/SIRT1 induced by compounds like resveratrol enhances nerve regeneration and axonal growth of ALS patient-derived mesenchymal stem cells [204]. Similarly, quercetin activates Sestrin2/AMPK/SIRT1 signaling pathway to reduce endoplasmic reticulum stress and alleviate apoptosis and inflammation in ALS as reviewed by Jin T. et al. [202].

- FGF-2

Fibroblast growth factor 2 (FGF-2), also known as basic fibroblast growth factor (bFGF), is a member of the fibroblast growth factor family, which includes proteins involved in various cellular processes such as cell growth, and differentiation. FGF-2 is expressed in various tissues and has been implicated in the pathogenesis of several diseases, including cancer, cardiovascular diseases, and neurological disorders like ALS [205].

FGF-2 contains four cysteine residues two of which, Cys 78 and Cys 96 are sensitive to redox changes. These cysteines play a role in the activation of FGF-2 through oligomerization and membrane pore formation, which involves the formation of disulfide bonds with other proteins or macromolecules [206].

Knocking down FGF-2 expression in ALS mouse models has been shown to delay disease onset and improve symptoms, suggesting its involvement in disease progression [207]. This suggests that FGF-2 may play a significant role in the ALS progression and could be a potential target for therapeutic interventions. Additional research is necessary to gain insight into the specific mechanisms by which FGF-2 influences ALS and to investigate its therapeutic potential more extensively.

## 5. Conclusions

Cysteine residues, with their unique thiol groups, are highly susceptible to oxidative modifications, which can significantly alter protein function, signaling pathways, and cellular homeostasis. The intricate relationship between cysteine PTMs and redox imbalance plays a crucial role in the pathogenesis of neurodegenerative diseases, particularly ALS. Evidence points to the dual role of cysteine PTMs in neurodegeneration. On one hand, reversible modifications, such as S-nitrosylation and S-glutathionylation, serve as protective mechanisms against irreversible oxidative damage. Conversely, aberrant modifications, such as S-sulfenylation and disulfide bond formation, can lead to loss of function, protein misfolding, and toxic aggregation, which are hallmarks of neurological conditions like ALS. Identifying the cysteine-modified proteins and elucidating the regulatory networks by which cysteine PTMs contribute to redox imbalance could be helpful for improving understanding of ALS and exploring potential new pharmacological interventions that modulate these signaling pathways.

Thousands of cysteine PTMs sites have been identified, and several cysteine PTM-based databases have been developed [208,209,210], including the recent CysModDB platform [89]. The identification of cysteine PTM sites on proteomes is the basis for exploring their functional roles in biological activities.

Redox proteomics, a powerful tool in this context, enables the identification and quantification of oxidative modifications on proteins, providing valuable insights into cellular mechanisms of redox environment. Technological advances in redox proteomics have significantly improved the ability to map these modifications with high precision, facilitating a deeper understanding of their biological impact in neurodegeneration [211,212,213].

Moreover, considering the interaction between PTMs and pathological mechanisms, such as inflammation, mitochondrial dysfunction, proteasomal impairment, and excitotoxicity, is crucial [214]. The close interaction between oxidative modifications and these cellular processes can exacerbate neurodegenerative diseases. PTMs can influence the activity of signaling proteins involved in inflammatory responses, further promoting neuroinflammation, a common feature of neurodegenerative diseases. Mitochondrial dysfunction, characterized by impaired energy production and increased OS, can be both a cause and consequence of altered PTMs. Proteasomal impairment, leading to the accumulation of misfolded and damaged proteins, is often linked with abnormal PTMs, which disrupt cellular homeostasis. Additionally, excitotoxicity, resulting from excessive glutamate signaling, can induce OS and influence PTM dynamics, thus contributing to neuronal damage [214].

Taking this tangled context into account, innovative pharmacological strategies can be designed. drawn [215]. Combined treatments that target multiple features of the pathological mechanisms simultaneously, can potentially provide more effective therapeutic outcomes.

For instance, PeaLut, which combines a lipid mediator with anti-inflammatory properties (Palmitoylethanolamide, Pea) and an antioxidant agent (Luteolin, Lut) has shown promising results in both animal models [216] and clinical studies [217] of neurodegenerative diseases, such as AD and FTD. The antioxidant properties of Lut may attenuate OS, while the anti-inflammatory effects of PEA may reduce neuroinflammation, offering a synergistic approach to managing neurodegenerative conditions.

Future research should continue to explore the dynamic landscape of PTMs, in particular Cys-PTMs, and their interactions with disease processes, as well as investigate the therapeutic potential of combined treatment strategies in clinical settings.

## Figures and Tables

**Table 1:** List of key cysteine modified proteins and their respective PTMs involved in major neurodegenerative diseases.

PTM	Protein	NDD
ROS-mediated		
Disulfide bond	SOD1	ALS [16]
	Tau	AD [17]
	TDP43	ALS, FTD [18]
Sulfinylation and sulfonation	DJ-1 (PARK7)	PD [19]
	Parkin	PD [20]
	SOD1	ALS [21]
	TTR	MS [22]
RNS-mediated		
S-nitrosylation	CDK5	AD [23]
	DJ-1	PD [24,25]
	DRP1	AD [26], HD [27]
	GAPDH	HD [28], PD [29]
	GOSPEL	PD [30]
	Parkin	PD [31]
	PDI	ALS [32]
	PRX2	PD [33]
	UCHL1	PD [34]
	XIAP	PD [35]
RSS-mediated		
S-sulfhydration (persulfidation)	Akt	AD [36]
	GSK3β	AD [37]
	Parkin	PD [38]
	TTR	MS [22,39]
	Sirt1	PD [40]

PTM, post-translational modification; NDD, neurodegenerative disorder; ROS, reactive oxygen species; RNS, reactive nitrogen species; RSS, reactive sulfur species. SOD1, superoxide dismutase 1; ALS, amyotrophic lateral sclerosis; AD, Alzheimer’s disease; TDP43, TAR DNA-binding protein 43; FTD, frontotemporal dementia; DJ1, protein deglycase DJ-1; PD, Parkinson’s disease; TTR, transthyretin; MS, multiple sclerosis; CDK5, cyclin-dependent kinase 5; DRP1, dynamin-related protein 1; HD, Huntington’s disease; GAPDH, glyceraldehyde 3-phosphate dehydrogenase; GOSPEL, GAPDH’s competitor of SIAH1 protein enhances life; PDI, protein disulfide isomerase; PRX2, peroxiredoxin 2; UCHL1; ubiquitin C-terminal hydrolase L1; XIAP, XIAP-X-linked inhibitor of apoptosis; Akt, protein kinase B; GSK3β, glycogen synthase kinase-3 beta; Sirt1, sirtuin-1.

## Data Availability

The data presented in this study are available in article.

## References

[1] Jennings E.Q., Fritz K.S., Galligan J.J. (2022). Biochemical genesis of enzymatic and non-enzymatic post-translational modifications. Mol. Aspects Med..

[2] Walsh C.T., Garneau-Tsodikova S., Gatto G.J. (2005). Protein Posttranslational Modifications: The Chemistry of Proteome Diversifications. Angew. Chemie Int. Ed..

[3] Ramazi S., Zahiri J. (2021). Post-translational modifications in proteins: Resources, tools and prediction methods. Database.

[4] Schaffert L.-N., Carter W.G. (2020). Do Post-Translational Modifications Influence Protein Aggregation in Neurodegenerative Diseases: A Systematic Review. Brain Sci..

[5] Martinez-Banaclocha M. (2022). N-Acetyl-Cysteine: Modulating the Cysteine Redox Proteome in Neurodegenerative Diseases. Antioxidants.

[6] Chung H.S., Wang S.-B., Venkatraman V., Murray C.I., Van Eyk J.E. (2013). Cysteine Oxidative Posttranslational Modifications. Circ. Res..

[7] Martínez Banaclocha M.A. (2016). Cellular Cysteine Network (CYSTEINET): Pharmacological Intervention in Brain Aging and Neurodegenerative Diseases. Frontiers in Clinical Drug Research-Central Nervous System.

[8] Poole L.B. (2015). The basics of thiols and cysteines in redox biology and chemistry. Free Radic. Biol. Med..

[9] Sbodio J.I., Snyder S.H., Paul B.D. (2019). Redox Mechanisms in Neurodegeneration: From Disease Outcomes to Therapeutic Opportunities. Antioxid. Redox Signal..

[10] Murphy M.P. (2009). How mitochondria produce reactive oxygen species. Biochem. J..

[11] Jones D.P. (2006). Redefining Oxidative Stress. Antioxid. Redox Signal..

[12] Floyd R.A., Carney J.M. (1992). Free radical damage to protein and DNA: Mechanisms involved and relevant observations on brain undergoing oxidative stress. Ann. Neurol..

[13] Islam M.T. (2017). Oxidative stress and mitochondrial dysfunction-linked neurodegenerative disorders. Neurol. Res..

[14] Greco V., Longone P., Spalloni A., Pieroni L., Urbani A. (2019). Crosstalk between Oxidative Stress and Mitochondrial Damage: Focus on Amyotrophic Lateral Sclerosis. Mitochondria in Health and in Sickness.

[15] Martínez-Banaclocha M. (2016). Cysteine Network (CYSTEINET) Dysregulation in Parkinson’s Disease: Role of N-acetylcysteine. Curr. Drug Metab..

[16] Bouldin S.D., Darch M.A., Hart P.J., Outten C.E. (2012). Redox properties of the disulfide bond of human Cu,Zn superoxide dismutase and the effects of human glutaredoxin 1. Biochem. J..

[17] Weismiller H.A., Holub T.J., Krzesinski B.J., Margittai M. (2021). A thiol-based intramolecular redox switch in four-repeat tau controls fibril assembly and disassembly. J. Biol. Chem..

[18] Cohen T.J., Hwang A.W., Unger T., Trojanowski J.Q., Lee V.M.Y. (2012). Redox signalling directly regulates TDP-43 via cysteine oxidation and disulphide cross-linking. EMBO J..

[19] Kiss R., Zhu M., Jójárt B., Czajlik A., Solti K., Fórizs B., Nagy É., Zsila F., Beke-Somfai T., Tóth G. (2017). Structural features of human DJ-1 in distinct Cys106 oxidative states and their relevance to its loss of function in disease. Biochim. Biophys. Acta-Gen. Subj..

[20] Meng F., Yao D., Shi Y., Kabakoff J., Wu W., Reicher J., Ma Y., Moosmann B., Masliah E., Lipton S.A. (2011). Oxidation of the cysteine-rich regions of parkin perturbs its E3 ligase activity and contributes to protein aggregation. Mol. Neurodegener..

[21] Chen X., Shang H., Qiu X., Fujiwara N., Cui L., Li X.-M., Gao T.-M., Kong J. (2012). Oxidative Modification of Cysteine 111 Promotes Disulfide Bond-Independent Aggregation of SOD1. Neurochem. Res..

[22] Pieragostino D., Del Boccio P., Di Ioia M., Pieroni L., Greco V., De Luca G., D’Aguanno S., Rossi C., Franciotta D., Centonze D. (2013). Oxidative modifications of cerebral transthyretin are associated with multiple sclerosis. Proteomics.

[23] Qu J., Nakamura T., Holland E.A., McKercher S.R., Lipton S.A. (2012). S-nitrosylation of Cdk5. Prion.

[24] Choi M.S., Nakamura T., Cho S.-J., Han X., Holland E.A., Qu J., Petsko G.A., Yates J.R., Liddington R.C., Lipton S.A. (2014). Transnitrosylation from DJ-1 to PTEN Attenuates Neuronal Cell Death in Parkinson’s Disease Models. J. Neurosci..

[25] Ito G., Ariga H., Nakagawa Y., Iwatsubo T. (2006). Roles of distinct cysteine residues in S-nitrosylation and dimerization of DJ-1. Biochem. Biophys. Res. Commun..

[26] Cho D.-H., Nakamura T., Fang J., Cieplak P., Godzik A., Gu Z., Lipton S.A. (2009). S-Nitrosylation of Drp1 Mediates β-Amyloid-Related Mitochondrial Fission and Neuronal Injury. Science.

[27] Haun F., Nakamura T., Shiu A.D., Cho D.-H., Tsunemi T., Holland E.A., La Spada A.R., Lipton S.A. (2013). S-Nitrosylation of Dynamin-Related Protein 1 Mediates Mutant Huntingtin-Induced Mitochondrial Fragmentation and Neuronal Injury in Huntington’s Disease. Antioxid. Redox Signal..

[28] Bae B.-I., Hara M.R., Cascio M.B., Wellington C.L., Hayden M.R., Ross C.A., Ha H.C., Li X.-J., Snyder S.H., Sawa A. (2006). Mutant Huntingtin: Nuclear translocation and cytotoxicity mediated by GAPDH. Proc. Natl. Acad. Sci. USA.

[29] Hara M.R., Thomas B., Cascio M.B., Bae B.-I., Hester L.D., Dawson V.L., Dawson T.M., Sawa A., Snyder S.H. (2006). Neuroprotection by pharmacologic blockade of the GAPDH death cascade. Proc. Natl. Acad. Sci. USA.

[30] Sen N., Hara M.R., Ahmad A.S., Cascio M.B., Kamiya A., Ehmsen J.T., Aggrawal N., Hester L., Doré S., Snyder S.H. (2009). GOSPEL: A Neuroprotective Protein that Binds to GAPDH upon S-Nitrosylation. Neuron.

[31] Yao D., Gu Z., Nakamura T., Shi Z.-Q., Ma Y., Gaston B., Palmer L.A., Rockenstein E.M., Zhang Z., Masliah E. (2004). Nitrosative stress linked to sporadic Parkinson’s disease: S-nitrosylation of parkin regulates its E3 ubiquitin ligase activity. Proc. Natl. Acad. Sci. USA.

[32] Chen X., Zhang X., Li C., Guan T., Shang H., Cui L., Li X., Kong J. (2013). S-nitrosylated protein disulfide isomerase contributes to mutant SOD1 aggregates in amyotrophic lateral sclerosis. J. Neurochem..

[33] Fang J., Nakamura T., Cho D.-H., Gu Z., Lipton S.A. (2007). S-nitrosylation of peroxiredoxin 2 promotes oxidative stress-induced neuronal cell death in Parkinson’s disease. Proc. Natl. Acad. Sci. USA.

[34] Kumar R., Jangir D.K., Verma G., Shekhar S., Hanpude P., Kumar S., Kumari R., Singh N., Bhavesh N.S., Jana N.R. (2017). S-nitrosylation of UCHL1 induces its structural instability and promotes α-synuclein aggregation. Sci. Rep..

[35] Tsang A.H.K., Lee Y.-I., Ko H.S., Savitt J.M., Pletnikova O., Troncoso J.C., Dawson V.L., Dawson T.M., Chung K.K.K. (2009). S-nitrosylation of XIAP compromises neuronal survival in Parkinson’s disease. Proc. Natl. Acad. Sci. USA.

[36] Sen T., Saha P., Jiang T., Sen N. (2020). Sulfhydration of AKT triggers Tau-phosphorylation by activating glycogen synthase kinase 3β in Alzheimer’s disease. Proc. Natl. Acad. Sci. USA.

[37] Giovinazzo D., Bursac B., Sbodio J.I., Nalluru S., Vignane T., Snowman A.M., Albacarys L.M., Sedlak T.W., Torregrossa R., Whiteman M. (2021). Hydrogen sulfide is neuroprotective in Alzheimer’s disease by sulfhydrating GSK3β and inhibiting Tau hyperphosphorylation. Proc. Natl. Acad. Sci. USA.

[38] Vandiver M.S., Paul B.D., Xu R., Karuppagounder S., Rao F., Snowman A.M., Seok Ko H., Il Lee Y., Dawson V.L., Dawson T.M. (2013). Sulfhydration mediates neuroprotective actions of parkin. Nat. Commun..

[39] Greco V., Neri C., Pieragostino D., Spalloni A., Persichilli S., Gastaldi M., Mercuri N.B., Longone P., Urbani A. (2021). Investigating Different Forms of Hydrogen Sulfide in Cerebrospinal Fluid of Various Neurological Disorders. Metabolites.

[40] Li J., Li M., Wang C., Zhang S., Gao Q., Wang L., Ma L. (2020). NaSH increases SIRT1 activity and autophagy flux through sulfhydration to protect SH-SY5Y cells induced by MPP~+. Cell Cycle.

[41] Alvarez B., Salinas G. (2022). Basic concepts of thiol chemistry and biology. Redox Chemistry and Biology of Thiols.

[42] Messens J., Collet J.-F. (2013). Thiol–Disulfide Exchange in Signaling: Disulfide Bonds As a Switch. Antioxid. Redox Signal..

[43] EREL Ö., ERDOĞAN S. (2020). Thiol-disulfide homeostasis: An integrated approach with biochemical and clinical aspects. TURKISH J. Med. Sci..

[44] McBean G.J., Aslan M., Griffiths H.R., Torrão R.C. (2015). Thiol redox homeostasis in neurodegenerative disease. Redox Biol..

[45] Arnesano F., Banci L., Bertini I., Martinelli M., Furukawa Y., O’Halloran T.V. (2004). The Unusually Stable Quaternary Structure of Human Cu,Zn-Superoxide Dismutase 1 Is Controlled by Both Metal Occupancy and Disulfide Status. J. Biol. Chem..

[46] Roos G., Messens J. (2011). Protein sulfenic acid formation: From cellular damage to redox regulation. Free Radic. Biol. Med..

[47] Lo Conte M., Carroll K.S. (2013). The Redox Biochemistry of Protein Sulfenylation and Sulfinylation. J. Biol. Chem..

[48] Beedle A.E.M., Lynham S., Garcia-Manyes S. (2016). Protein S-sulfenylation is a fleeting molecular switch that regulates non-enzymatic oxidative folding. Nat. Commun..

[49] Peskin A.V., Dickerhof N., Poynton R.A., Paton L.N., Pace P.E., Hampton M.B., Winterbourn C.C. (2013). Hyperoxidation of Peroxiredoxins 2 and 3. J. Biol. Chem..

[50] Biteau B., Labarre J., Toledano M.B. (2003). ATP-dependent reduction of cysteine–sulphinic acid by S. cerevisiae sulphiredoxin. Nature.

[51] Yang K.-S., Kang S.W., Woo H.A., Hwang S.C., Chae H.Z., Kim K., Rhee S.G. (2002). Inactivation of Human Peroxiredoxin I during Catalysis as the Result of the Oxidation of the Catalytic Site Cysteine to Cysteine-sulfinic Acid. J. Biol. Chem..

[52] Di Meo S., Reed T.T., Venditti P., Victor V.M. (2016). Role of ROS and RNS Sources in Physiological and Pathological Conditions. Oxid. Med. Cell. Longev..

[53] Bian K. (2003). Nitric oxide NO–biogeneration regulation and relevence to human diseases. Front. Biosci..

[54] Radi R. (2013). Peroxynitrite, a Stealthy Biological Oxidant. J. Biol. Chem..

[55] Kirsch M., Korth H.-G., Sustmann R., Groot H. (2002). de The Pathobiochemistry of Nitrogen Dioxide. Biol. Chem..

[56] Anand P., Stamler J.S. (2012). Enzymatic mechanisms regulating protein S-nitrosylation: Implications in health and disease. J. Mol. Med..

[57] Sun J., Steenbergen C., Murphy E. (2006). *S*-Nitrosylation: NO-Related Redox Signaling to Protect Against Oxidative Stress. Antioxid. Redox Signal..

[58] Seth D., Stamler J.S. (2011). The SNO-proteome: Causation and classifications. Curr. Opin. Chem. Biol..

[59] Koriyama Y., Furukawa A. (2018). S-Nitrosylation Regulates Cell Survival and Death in the Central Nervous System. Neurochem. Res..

[60] Fernando V., Zheng X., Walia Y., Sharma V., Letson J., Furuta S. (2019). S-Nitrosylation: An Emerging Paradigm of Redox Signaling. Antioxidants.

[61] Hess D.T., Matsumoto A., Kim S.-O., Marshall H.E., Stamler J.S. (2005). Protein S-nitrosylation: Purview and parameters. Nat. Rev. Mol. Cell Biol..

[62] Bouillaud F., Blachier F. (2011). Mitochondria and Sulfide: A Very Old Story of Poisoning, Feeding, and Signaling?. Antioxid. Redox Signal..

[63] Iciek M., Bilska-Wilkosz A., Kozdrowicki M., Górny M. (2022). Reactive sulfur species and their significance in health and disease. Biosci. Rep..

[64] Libiad M., Yadav P.K., Vitvitsky V., Martinov M., Banerjee R. (2014). Organization of the Human Mitochondrial Hydrogen Sulfide Oxidation Pathway. J. Biol. Chem..

[65] Kabil O., Banerjee R. (2014). Enzymology of H_2_S Biogenesis, Decay and Signaling. Antioxid. Redox Signal..

[66] Shibuya N., Tanaka M., Yoshida M., Ogasawara Y., Togawa T., Ishii K., Kimura H. (2009). 3-Mercaptopyruvate Sulfurtransferase Produces Hydrogen Sulfide and Bound Sulfane Sulfur in the Brain. Antioxid. Redox Signal..

[67] Shibuya N., Koike S., Tanaka M., Ishigami-Yuasa M., Kimura Y., Ogasawara Y., Fukui K., Nagahara N., Kimura H. (2013). A novel pathway for the production of hydrogen sulfide from D-cysteine in mammalian cells. Nat. Commun..

[68] Mustafa A.K., Gadalla M.M., Sen N., Kim S., Mu W., Gazi S.K., Barrow R.K., Yang G., Wang R., Snyder S.H. (2009). H_2_S Signals through Protein S-Sulfhydration. Sci. Signal..

[69] Paul B.D., Snyder S.H. (2012). H2S signalling through protein sulfhydration and beyond. Nat. Rev. Mol. Cell Biol..

[70] Paul B.D., Snyder S.H. (2015). H 2 S: A Novel Gasotransmitter that Signals by Sulfhydration. Trends Biochem. Sci..

[71] Zhang D., Du J., Tang C., Huang Y., Jin H. (2017). H_2_S-Induced Sulfhydration: Biological Function and Detection Methodology. Front. Pharmacol..

[72] Petrovic D., Kouroussis E., Vignane T., Filipovic M.R. (2021). The Role of Protein Persulfidation in Brain Aging and Neurodegeneration. Front. Aging Neurosci..

[73] Cuevasanta E., Lange M., Bonanata J., Coitiño E.L., Ferrer-Sueta G., Filipovic M.R., Alvarez B. (2015). Reaction of Hydrogen Sulfide with Disulfide and Sulfenic Acid to Form the Strongly Nucleophilic Persulfide. J. Biol. Chem..

[74] Szabo C. (2021). Hydrogen Sulfide, an Endogenous Stimulator of Mitochondrial Function in Cancer Cells. Cells.

[75] Cirino G., Szabo C., Papapetropoulos A. (2023). Physiological roles of hydrogen sulfide in mammalian cells, tissues, and organs. Physiol. Rev..

[76] Davoli A., Greco V., Spalloni A., Guatteo E., Neri C., Rizzo G.R., Cordella A., Romigi A., Cortese C., Bernardini S. (2015). Evidence of hydrogen sulfide involvement in amyotrophic lateral sclerosis. Ann. Neurol..

[77] Spalloni A., Greco V., Ciriminna G., Corasolla Carregari V., Marini F., Pieroni L., Mercuri N.B., Urbani A., Longone P. (2019). Impact of Pharmacological Inhibition of Hydrogen Sulphide Production in the SOD1G93A-ALS Mouse Model. Int. J. Mol. Sci..

[78] Kamoun P., Belardinelli M., Chabli A., Lallouchi K., Chadefaux-Vekemans B. (2003). Endogenous hydrogen sulfide overproduction in Down syndrome. Am. J. Med. Genet. Part A.

[79] Belardinelli M.-C., Chabli A., Chadefaux-Vekemans B., Kamoun P. (2001). Urinary Sulfur Compounds in Down Syndrome. Clin. Chem..

[80] Barayeu U., Sawa T., Nishida M., Wei F., Motohashi H., Akaike T. (2023). Supersulfide biology and translational medicine for disease control. Br. J. Pharmacol..

[81] Wu Z., Barayeu U., Schilling D., Dick T.P., Pratt D.A. (2023). Emergence of (hydro)persulfides as suppressors of lipid peroxidation and ferroptotic cell death. Curr. Opin. Chem. Biol..

[82] Nagy P., Winterbourn C.C. (2010). Rapid Reaction of Hydrogen Sulfide with the Neutrophil Oxidant Hypochlorous Acid to Generate Polysulfides. Chem. Res. Toxicol..

[83] Kimura Y., Mikami Y., Osumi K., Tsugane M., Oka J., Kimura H. (2013). Polysulfides are possible H_2_S-derived signaling molecules in rat brain. FASEB J..

[84] Kimura H. (2016). Hydrogen polysulfide (H_2_S*_n_*) signaling along with hydrogen sulfide (H_2_S) and nitric oxide (NO). J. Neural Transm..

[85] Greiner R., Pálinkás Z., Bäsell K., Becher D., Antelmann H., Nagy P., Dick T.P. (2013). Polysulfides Link H_2_S to Protein Thiol Oxidation. Antioxid. Redox Signal..

[86] Olson K.R. (2018). H_2_S and polysulfide metabolism: Conventional and unconventional pathways. Biochem. Pharmacol..

[87] Bogdándi V., Ditrói T., Bátai I.Z., Sándor Z., Minnion M., Vasas A., Galambos K., Buglyó P., Pintér E., Feelisch M. (2020). Nitrosopersulfide (SSNO^−^) Is a Unique Cysteine Polysulfidating Agent with Reduction-Resistant Bioactivity. Antioxid. Redox Signal..

[88] Alcock L.J., Perkins M.V., Chalker J.M. (2018). Chemical methods for mapping cysteine oxidation. Chem. Soc. Rev..

[89] Meng Y., Zhang L., Zhang L., Wang Z., Wang X., Li C., Chen Y., Shang S., Li L. (2022). CysModDB: A comprehensive platform with the integration of manually curated resources and analysis tools for cysteine posttranslational modifications. Brief. Bioinform..

[90] Gallogly M.M., Mieyal J.J. (2007). Mechanisms of reversible protein glutathionylation in redox signaling and oxidative stress. Curr. Opin. Pharmacol..

[91] Dalle-Donne I., Rossi R., Giustarini D., Colombo R., Milzani A. (2007). S-glutathionylation in protein redox regulation. Free Radic. Biol. Med..

[92] Hurd T.R., Costa N.J., Dahm C.C., Beer S.M., Brown S.E., Filipovska A., Murphy M.P. (2005). Glutathionylation of Mitochondrial Proteins. Antioxid. Redox Signal..

[93] Applegate M.A.B., Humphries K.M., Szweda L.I. (2008). Reversible Inhibition of α-Ketoglutarate Dehydrogenase by Hydrogen Peroxide:  Glutathionylation and Protection of Lipoic Acid. Biochemistry.

[94] Bulteau A.-L., Lundberg K.C., Ikeda-Saito M., Isaya G., Szweda L.I. (2005). Reversible redox-dependent modulation of mitochondrial aconitase and proteolytic activity during in vivo cardiac ischemia/reperfusion. Proc. Natl. Acad. Sci. USA.

[95] Giangregorio N., Palmieri F., Indiveri C. (2013). Glutathione controls the redox state of the mitochondrial carnitine/acylcarnitine carrier Cys residues by glutathionylation. Biochim. Biophys. Acta-Gen. Subj..

[96] Willems P.H.G.M., Rossignol R., Dieteren C.E.J., Murphy M.P., Koopman W.J.H. (2015). Redox Homeostasis and Mitochondrial Dynamics. Cell Metab..

[97] Vrettou S., Wirth B. (2022). S-Glutathionylation and S-Nitrosylation in Mitochondria: Focus on Homeostasis and Neurodegenerative Diseases. Int. J. Mol. Sci..

[98] Cha S.J., Kim H., Choi H.-J., Lee S., Kim K. (2017). Protein Glutathionylation in the Pathogenesis of Neurodegenerative Diseases. Oxid. Med. Cell. Longev..

[99] Newman S.F., Sultana R., Perluigi M., Coccia R., Cai J., Pierce W.M., Klein J.B., Turner D.M., Butterfield D.A. (2007). An increase in S-glutathionylated proteins in the Alzheimer’s disease inferior parietal lobule, a proteomics approach. J. Neurosci. Res..

[100] Dinoto L., Deture M.A., Purich D.L. (2005). Structural insights into Alzheimer filament assembly pathways based on site-directed mutagenesis and *S*-glutathionylation of three-repeat neuronal Tau protein. Microsc. Res. Tech..

[101] Hutson S.M., Wallin R., Hall T.R. (1992). Identification of mitochondrial branched chain aminotransferase and its isoforms in rat tissues. J. Biol. Chem..

[102] El Kodsi D.N., Tokarew J.M., Sengupta R., Lengacher N.A., Chatterji A., Nguyen A.P., Boston H., Jiang Q., Palmberg C., Pileggi C. (2023). Parkin coregulates glutathione metabolism in adult mammalian brain. Acta Neuropathol. Commun..

[103] Johnson W.M., Golczak M., Choe K., Curran P.L., Miller O.G., Yao C., Wang W., Lin J., Milkovic N.M., Ray A. (2016). Regulation of DJ-1 by Glutaredoxin 1 in Vivo: Implications for Parkinson’s Disease. Biochemistry.

[104] Redler R.L., Wilcox K.C., Proctor E.A., Fee L., Caplow M., Dokholyan N.V. (2011). Glutathionylation at Cys-111 Induces Dissociation of Wild Type and FALS Mutant SOD1 Dimers. Biochemistry.

[105] Lee J., Ryu H., Kowall N.W. (2009). Differential regulation of neuronal and inducible nitric oxide synthase (NOS) in the spinal cord of mutant SOD1 (G93A) ALS mice. Biochem. Biophys. Res. Commun..

[106] Viappiani S., Nicolescu A.C., Holt A., Sawicki G., Crawford B.D., León H., van Mulligen T., Schulz R. (2009). Activation and modulation of 72 kDa matrix metalloproteinase-2 by peroxynitrite and glutathione. Biochem. Pharmacol..

[107] Kar S., Subbaram S., Carrico P.M., Melendez J.A. (2010). Redox-control of matrix metalloproteinase-1: A critical link between free radicals, matrix remodeling and degenerative disease. Respir. Physiol. Neurobiol..

[108] Dalle-Donne I., Giustarini D., Colombo R., Rossi R., Milzani A. (2003). Protein carbonylation in human diseases. Trends Mol. Med..

[109] Reed T.T., Butterfield D.A. (2017). Protein Carbonylation in Brains of Subjects with Selected Neurodegenerative Disorders. Protein Carbonylation.

[110] Sharma A., Weber D., Raupbach J., Dakal T.C., Fließbach K., Ramirez A., Grune T., Wüllner U. (2020). Advanced glycation end products and protein carbonyl levels in plasma reveal sex-specific differences in Parkinson’s and Alzheimer’s disease. Redox Biol..

[111] Sadowska-Bartosz I., Adamczyk-Sowa M., Galiniak S., Mucha S., Pierzchala K., Bartosz G. (2013). Oxidative modification of serum proteins in multiple sclerosis. Neurochem. Int..

[112] Chamberlain L.H., Shipston M.J. (2015). The Physiology of Protein *S*-acylation. Physiol. Rev..

[113] Mesquita F.S., Abrami L., Linder M.E., Bamji S.X., Dickinson B.C., van der Goot F.G. (2024). Mechanisms and functions of protein S-acylation. Nat. Rev. Mol. Cell Biol..

[114] Conibear E., Davis N.G. (2010). Palmitoylation and depalmitoylation dynamics at a glance. J. Cell Sci..

[115] Hornemann T. (2015). Palmitoylation and depalmitoylation defects. J. Inherit. Metab. Dis..

[116] Ramzan F., Abrar F., Mishra G.G., Liao L.M.Q., Martin D.D.O. (2023). Lost in traffic: Consequences of altered palmitoylation in neurodegeneration. Front. Physiol..

[117] Fukata Y., Fukata M. (2010). Protein palmitoylation in neuronal development and synaptic plasticity. Nat. Rev. Neurosci..

[118] Sanders S.S., Martin D.D.O., Butland S.L., Lavallée-Adam M., Calzolari D., Kay C., Yates J.R., Hayden M.R. (2015). Curation of the Mammalian Palmitoylome Indicates a Pivotal Role for Palmitoylation in Diseases and Disorders of the Nervous System and Cancers. PLoS Comput. Biol..

[119] Yanai A., Huang K., Kang R., Singaraja R.R., Arstikaitis P., Gan L., Orban P.C., Mullard A., Cowan C.M., Raymond L.A. (2006). Palmitoylation of huntingtin by HIP14is essential for its trafficking and function. Nat. Neurosci..

[120] Bhattacharyya R., Barren C., Kovacs D.M. (2013). Palmitoylation of Amyloid Precursor Protein Regulates Amyloidogenic Processing in Lipid Rafts. J. Neurosci..

[121] Cheng H., Vetrivel K.S., Drisdel R.C., Meckler X., Gong P., Leem J.Y., Li T., Carter M., Chen Y., Nguyen P. (2009). S-Palmitoylation of γ-Secretase Subunits Nicastrin and APH-1. J. Biol. Chem..

[122] Koegl M., Zlatkine P., Ley S.C., Courtneidge S.A., Magee A.I. (1994). Palmitoylation of multiple Src-family kinases at a homologous N-terminal motif. Biochem. J..

[123] Antinone S.E., Ghadge G.D., Lam T.T., Wang L., Roos R.P., Green W.N. (2013). Palmitoylation of Superoxide Dismutase 1 (SOD1) Is Increased for Familial Amyotrophic Lateral Sclerosis-linked SOD1 Mutants. J. Biol. Chem..

[124] Jung D., Bachmann H.S. (2023). Regulation of protein prenylation. Biomed. Pharmacother..

[125] Zhang F.L., Casey P.J. (1996). PROTEIN PRENYLATION: Molecular Mechanisms and Functional Consequences. Annu. Rev. Biochem..

[126] Xu N., Shen N., Wang X., Jiang S., Xue B., Li C. (2015). Protein prenylation and human diseases: A balance of protein farnesylation and geranylgeranylation. Sci. China Life Sci..

[127] Cole S.L., Vassar R. (2006). Isoprenoids and Alzheimer’s disease: A complex relationship. Neurobiol. Dis..

[128] Eckert G.P., Hooff G.P., Strandjord D.M., Igbavboa U., Volmer D.A., Müller W.E., Wood W.G. (2009). Regulation of the brain isoprenoids farnesyl- and geranylgeranylpyrophosphate is altered in male Alzheimer patients. Neurobiol. Dis..

[129] Hooff G.P., Peters I., Wood W.G., Müller W.E., Eckert G.P. (2010). Modulation of Cholesterol, Farnesylpyrophosphate, and Geranylgeranylpyrophosphate in Neuroblastoma SH-SY5Y-APP695 Cells: Impact on Amyloid Beta-Protein Production. Mol. Neurobiol..

[130] Ma H., Huo J., Xin C., Yang J., Liu Q., Dong H., Li R., Liu Y. (2024). RABGGTB plays a critical role in ALS pathogenesis. Brain Res. Bull..

[131] Jo A., Lee Y., Kam T.-I., Kang S.-U., Neifert S., Karuppagounder S.S., Khang R., Kang H., Park H., Chou S.-C. (2021). PARIS farnesylation prevents neurodegeneration in models of Parkinson’s disease. Sci. Transl. Med..

[132] Chen S.-M., Tang X.-Q. (2022). Homocysteinylation and Sulfhydration in Diseases. Curr. Neuropharmacol..

[133] Jakubowski H. (2004). Molecular basis of homocysteine toxicity in humans. Cell. Mol. Life Sci..

[134] Sass J.O., Nakanishi T., Sato T., Sperl W., Shimizu A. (2003). S-Homocysteinylation of transthyretin is detected in plasma and serum of humans with different types of hyperhomocysteinemia. Biochem. Biophys. Res. Commun..

[135] Carey A., Fossati S. (2023). Hypertension and hyperhomocysteinemia as modifiable risk factors for Alzheimer’s disease and dementia: New evidence, potential therapeutic strategies, and biomarkers. Alzheimer’s Dement..

[136] Cortes-Canteli M., Paul J., Norris E.H., Bronstein R., Ahn H.J., Zamolodchikov D., Bhuvanendran S., Fenz K.M., Strickland S. (2010). Fibrinogen and β-Amyloid Association Alters Thrombosis and Fibrinolysis: A Possible Contributing Factor to Alzheimer’s Disease. Neuron.

[137] Chung Y.C., Kruyer A., Yao Y., Feierman E., Richards A., Strickland S., Norris E.H. (2016). Hyperhomocysteinemia exacerbates Alzheimer’s disease pathology by way of the β-amyloid fibrinogen interaction. J. Thromb. Haemost..

[138] Riquier S., Breton J., Abbas K., Cornu D., Bouton C., Drapier J.-C. (2014). Peroxiredoxin post-translational modifications by redox messengers. Redox Biol..

[139] McDowell G.S., Philpott A. (2016). New Insights into the Role of Ubiquitylation of Proteins. Int. Rev. Cell Mol. Biol..

[140] McDowell G.S., Philpott A. (2013). Non-canonical ubiquitylation: Mechanisms and consequences. Int. J. Biochem. Cell Biol..

[141] McClellan A.J., Laugesen S.H., Ellgaard L. (2019). Cellular functions and molecular mechanisms of non-lysine ubiquitination. Open Biol..

[142] Sabatelli M., Marangi G., Conte A., Tasca G., Zollino M., Lattante S. (2016). New ALS-Related Genes Expand the *Spectrum Paradigm* of Amyotrophic Lateral Sclerosis. Brain Pathol..

[143] Barber S.C., Shaw P.J. (2010). Oxidative stress in ALS: Key role in motor neuron injury and therapeutic target. Free Radic. Biol. Med..

[144] Jankovic M., Novakovic I., Gamil Anwar Dawod P., Gamil Anwar Dawod A., Drinic A., Abdel Motaleb F.I., Ducic S., Nikolic D. (2021). Current Concepts on Genetic Aspects of Mitochondrial Dysfunction in Amyotrophic Lateral Sclerosis. Int. J. Mol. Sci..

[145] Jagaraj C.J., Parakh S., Atkin J.D. (2021). Emerging Evidence Highlighting the Importance of Redox Dysregulation in the Pathogenesis of Amyotrophic Lateral Sclerosis (ALS). Front. Cell. Neurosci..

[146] Valle C., Carrì M.T. (2017). Cysteine Modifications in the Pathogenesis of ALS. Front. Mol. Neurosci..

[147] Meister A. (1988). Glutathione metabolism and its selective modification. J. Biol. Chem..

[148] Forman H.J. (2016). Redox signaling: An evolution from free radicals to aging. Free Radic. Biol. Med..

[149] Golko-Perez S., Amit T., Bar-Am O., Youdim M.B.H., Weinreb O. (2017). A Novel Iron Chelator-Radical Scavenger Ameliorates Motor Dysfunction and Improves Life Span and Mitochondrial Biogenesis in SOD1G93A ALS Mice. Neurotox. Res..

[150] Petillon C., Hergesheimer R., Puy H., Corcia P., Vourc’h P., Andres C., Karim Z., Blasco H. (2019). The relevancy of data regarding the metabolism of iron to our understanding of deregulated mechanisms in ALS; hypotheses and pitfalls. Front. Neurosci..

[151] Silva-Adaya D., Gonsebatt M.E., Guevara J. (2014). Thioredoxin System Regulation in the Central Nervous System: Experimental Models and Clinical Evidence. Oxid. Med. Cell. Longev..

[152] Chae H.Z., Kim H.J., Kang S.W., Rhee S.G. (1999). Characterization of three isoforms of mammalian peroxiredoxin that reduce peroxides in the presence of thioredoxin. Diabetes Res. Clin. Pract..

[153] Lu J., Holmgren A. (2014). The thioredoxin antioxidant system. Free Radic. Biol. Med..

[154] Malaspina A., Kaushik N., De Belleroche J. (2001). Differential expression of 14 genes in amyotrophic lateral sclerosis spinal cord detected using gridded cDNA arrays. J. Neurochem..

[155] Ogawa Y., Kosaka H., Nakanishi T., Shimizu A., Ohoi N., Shouji H., Yanagihara T., Sakoda S. (1997). Stability of Mutant Superoxide Dismutase-1 Associated with Familial Amyotrophic Lateral Sclerosis Determines the Manner of Copper Release and Induction of Thioredoxin in Erythrocytes. Biochem. Biophys. Res. Commun..

[156] Asensi M., Sastre J., Pallardo F.V., Lloret A., Lehner M., Garcia-de-la Asuncion J., Viña J. (1999). [23] Ratio of reduced to oxidized glutathione as indicator of oxidative stress status and DNA damage. Methods Enzymol..

[157] Ehrhart J., Smith A.J., Kuzmin-Nichols N., Zesiewicz T.A., Jahan I., Shytle R.D., Kim S.-H., Sanberg C.D., Vu T.H., Gooch C.L. (2015). Humoral factors in ALS patients during disease progression. J. Neuroinflamm..

[158] Chi L., Ke Y., Luo C., Gozal D., Liu R. (2007). Depletion of reduced glutathione enhances motor neuron degeneration in vitro and in vivo. Neuroscience.

[159] Rhee S.G., Chae H.Z., Kim K. (2005). Peroxiredoxins: A historical overview and speculative preview of novel mechanisms and emerging concepts in cell signaling. Free Radic. Biol. Med..

[160] Wood Z.A., Schröder E., Robin Harris J., Poole L.B. (2003). Structure, mechanism and regulation of peroxiredoxins. Trends Biochem. Sci..

[161] Rhee S.G. (2016). Overview on Peroxiredoxin. Mol. Cells.

[162] Szeliga M. (2020). Peroxiredoxins in Neurodegenerative Diseases. Antioxidants.

[163] Wood-Allum C.A. (2006). Impairment of mitochondrial anti-oxidant defence in SOD1-related motor neuron injury and amelioration by ebselen. Brain.

[164] Kriznik A., Libiad M., Le Cordier H., Boukhenouna S., Toledano M.B., Rahuel-Clermont S. (2020). Dynamics of a Key Conformational Transition in the Mechanism of Peroxiredoxin Sulfinylation. ACS Catal..

[165] Strey C.W., Spellman D., Stieber A., Gonatas J.O., Wang X., Lambris J.D., Gonatas N.K. (2004). Dysregulation of Stathmin, a Microtubule-Destabilizing Protein, and Up-Regulation of Hsp25, Hsp27, and the Antioxidant Peroxiredoxin 6 in a Mouse Model of Familial Amyotrophic Lateral Sclerosis. Am. J. Pathol..

[166] Allen S., Heath P.R., Kirby J., Wharton S.B., Cookson M.R., Menzies F.M., Banks R.E., Shaw P.J. (2003). Analysis of the Cytosolic Proteome in a Cell Culture Model of Familial Amyotrophic Lateral Sclerosis Reveals Alterations to the Proteasome, Antioxidant Defenses, and Nitric Oxide Synthetic Pathways. J. Biol. Chem..

[167] Devos D., Moreau C., Kyheng M., Garçon G., Rolland A.S., Blasco H., Gelé P., Lenglet T.T., Veyrat-Durebex C., Corcia P. (2019). Author Correction: A ferroptosis–based panel of prognostic biomarkers for Amyotrophic Lateral Sclerosis. Sci. Rep..

[168] Kawamata H., Manfredi G. (2008). Different regulation of wild-type and mutant Cu,Zn superoxide dismutase localization in mammalian mitochondria. Hum. Mol. Genet..

[169] Culotta V.C., Klomp L.W.J., Strain J., Casareno R.L.B., Krems B., Gitlin J.D. (1997). The Copper Chaperone for Superoxide Dismutase. J. Biol. Chem..

[170] Kawamata H., Manfredi G. (2010). Import, maturation, and function of SOD1 and its copper chaperone CCS in the mitochondrial intermembrane space. Antioxid. Redox Signal..

[171] Harraz M.M., Marden J.J., Zhou W., Zhang Y., Williams A., Sharov V.S., Nelson K., Luo M., Paulson H., Schöneich C. (2008). SOD1 mutations disrupt redox-sensitive Rac regulation of NADPH oxidase in a familial ALS model. J. Clin. Investig..

[172] Tafuri F., Ronchi D., Magri F., Comi G.P., Corti S. (2015). SOD1 misplacing and mitochondrial dysfunction in amyotrophic lateral sclerosis pathogenesis. Front. Cell. Neurosci..

[173] Sasaki S., Warita H., Murakami T., Shibata N., Komori T., Abe K., Kobayashi M., Iwata M. (2005). Ultrastructural study of aggregates in the spinal cord of transgenic mice with a G93A mutant SOD1 gene. Acta Neuropathol..

[174] Higgins C.M.J., Jung C., Ding H., Xu Z. (2002). Mutant Cu, Zn Superoxide Dismutase that Causes Motoneuron Degeneration Is Present in Mitochondria in the CNS. J. Neurosci..

[175] McAlary L., Yerbury J.J., Aquilina J.A. (2013). Glutathionylation potentiates benign superoxide dismutase 1 variants to the toxic forms associated with amyotrophic lateral sclerosis. Sci. Rep..

[176] Buratti E. (2018). TDP-43 post-translational modifications in health and disease. Expert Opin. Ther. Targets.

[177] Bhardwaj A., Myers M.P., Buratti E., Baralle F.E. (2013). Characterizing TDP-43 interaction with its RNA targets. Nucleic Acids Res..

[178] Prasad A., Bharathi V., Sivalingam V., Girdhar A., Patel B.K. (2019). Molecular Mechanisms of TDP-43 Misfolding and Pathology in Amyotrophic Lateral Sclerosis. Front. Mol. Neurosci..

[179] Igaz L.M., Kwong L.K., Xu Y., Truax A.C., Uryu K., Neumann M., Clark C.M., Elman L.B., Miller B.L., Grossman M. (2008). Enrichment of C-Terminal Fragments in TAR DNA-Binding Protein-43 Cytoplasmic Inclusions in Brain but not in Spinal Cord of Frontotemporal Lobar Degeneration and Amyotrophic Lateral Sclerosis. Am. J. Pathol..

[180] Bozzo F., Salvatori I., Iacovelli F., Mirra A., Rossi S., Cozzolino M., Falconi M., Valle C., Carrì M.T. (2016). Structural insights into the multi-determinant aggregation of TDP-43 in motor neuron-like cells. Neurobiol. Dis..

[181] Deng H., Gao K., Jankovic J. (2014). The role of FUS gene variants in neurodegenerative diseases. Nat. Rev. Neurol..

[182] Da Cruz S., Cleveland D.W. (2011). Understanding the role of TDP-43 and FUS/TLS in ALS and beyond. Curr. Opin. Neurobiol..

[183] Dormann D., Rodde R., Edbauer D., Bentmann E., Fischer I., Hruscha A., Than M.E., Mackenzie I.R.A., Capell A., Schmid B. (2010). ALS-associated fused in sarcoma (FUS) mutations disrupt Transportin-mediated nuclear import. EMBO J..

[184] Deng Q., Holler C.J., Taylor G., Hudson K.F., Watkins W., Gearing M., Ito D., Murray M.E., Dickson D.W., Seyfried N.T. (2014). FUS is Phosphorylated by DNA-PK and Accumulates in the Cytoplasm after DNA Damage. J. Neurosci..

[185] Monahan Z., Ryan V.H., Janke A.M., Burke K.A., Rhoads S.N., Zerze G.H., O’Meally R., Dignon G.L., Conicella A.E., Zheng W. (2017). Phosphorylation of the FUS low-complexity domain disrupts phase separation, aggregation, and toxicity. EMBO J..

[186] Cha S.J., Lee S., Choi H.-J., Han Y.J., Jeon Y.-M., Jo M., Lee S., Nahm M., Lim S.M., Kim S.H. (2022). Therapeutic modulation of GSTO activity rescues FUS-associated neurotoxicity via deglutathionylation in ALS disease models. Dev. Cell.

[187] Walker A.K. (2010). Protein Disulfide Isomerase and the Endoplasmic Reticulum in Amyotrophic Lateral Sclerosis. J. Neurosci..

[188] Parakh S., Shadfar S., Perri E.R., Ragagnin A.M.G., Piattoni C.V., Fogolín M.B., Yuan K.C., Shahheydari H., Don E.K., Thomas C.J. (2020). The Redox Activity of Protein Disulfide Isomerase Inhibits ALS Phenotypes in Cellular and Zebrafish Models. iScience.

[189] Rozas P., Pinto C., Martínez Traub F., Díaz R., Pérez V., Becerra D., Ojeda P., Ojeda J., Wright M.T., Mella J. (2021). Protein disulfide isomerase ERp57 protects early muscle denervation in experimental ALS. Acta Neuropathol. Commun..

[190] Paulsen C.E., Carroll K.S. (2013). Cysteine-Mediated Redox Signaling: Chemistry, Biology, and Tools for Discovery. Chem. Rev..

[191] Fra A., Yoboue E.D., Sitia R. (2017). Cysteines as Redox Molecular Switches and Targets of Disease. Front. Mol. Neurosci..

[192] Farg M.A., Soo K.Y., Walker A.K., Pham H., Orian J., Horne M.K., Warraich S.T., Williams K.L., Blair I.P., Atkin J.D. (2012). Mutant FUS induces endoplasmic reticulum stress in amyotrophic lateral sclerosis and interacts with protein disulfide-isomerase. Neurobiol. Aging.

[193] Jeon G.S., Nakamura T., Lee J.-S., Choi W.-J., Ahn S.-W., Lee K.-W., Sung J.-J., Lipton S.A. (2014). Potential Effect of S-Nitrosylated Protein Disulfide Isomerase on Mutant SOD1 Aggregation and Neuronal Cell Death in Amyotrophic Lateral Sclerosis. Mol. Neurobiol..

[194] Schonhoff C.M., Matsuoka M., Tummala H., Johnson M.A., Estevéz A.G., Wu R., Kamaid A., Ricart K.C., Hashimoto Y., Gaston B. (2006). *S*-nitrosothiol depletion in amyotrophic lateral sclerosis. Proc. Natl. Acad. Sci. USA.

[195] Liu Y.-J., Chern Y. (2021). Contribution of Energy Dysfunction to Impaired Protein Translation in Neurodegenerative Diseases. Front. Cell. Neurosci..

[196] Hinchy E.C., Gruszczyk A.V., Willows R., Navaratnam N., Hall A.R., Bates G., Bright T.P., Krieg T., Carling D., Murphy M.P. (2018). Mitochondria-derived ROS activate AMP-activated protein kinase (AMPK) indirectly. J. Biol. Chem..

[197] Zmijewski J.W., Banerjee S., Bae H., Friggeri A., Lazarowski E.R., Abraham E. (2010). Exposure to Hydrogen Peroxide Induces Oxidation and Activation of AMP-activated Protein Kinase*. J. Biol. Chem..

[198] Shao D., Oka S., Liu T., Zhai P., Ago T., Sciarretta S., Li H., Sadoshima J. (2014). A Redox-Dependent Mechanism for Regulation of AMPK Activation by Thioredoxin1 during Energy Starvation. Cell Metab..

[199] Lim M.A., Selak M.A., Xiang Z., Krainc D., Neve R.L., Kraemer B.C., Watts J.L., Kalb R.G. (2012). Reduced Activity of AMP-Activated Protein Kinase Protects against Genetic Models of Motor Neuron Disease. J. Neurosci..

[200] Liu Y.-J., Ju T.-C., Chen H.-M., Jang Y.-S., Lee L.-M., Lai H.-L., Tai H.-C., Fang J.-M., Lin Y.-L., Tu P.-H. (2015). Activation of AMP-activated protein kinase α1 mediates mislocalization of TDP-43 in amyotrophic lateral sclerosis. Hum. Mol. Genet..

[201] Yuan M., Yan R., Zhang Y., Qiu Y., Jiang Z., Liu H., Wang Y., Sun L., Zhang H., Gao P. (2021). CARS senses cysteine deprivation to activate AMPK for cell survival. EMBO J..

[202] Jin T., Zhang Y., Botchway B.O.A., Huang M., Lu Q., Liu X. (2023). Quercetin activates the Sestrin2/AMPK/SIRT1 axis to improve amyotrophic lateral sclerosis. Biomed. Pharmacother..

[203] Ruderman N.B., Julia Xu X., Nelson L., Cacicedo J.M., Saha A.K., Lan F., Ido Y. (2010). AMPK and SIRT1: A long-standing partnership?. Am. J. Physiol. Metab..

[204] Yun Y.C., Jeong S., Kim S.H., Cho G. (2018). Reduced sirtuin 1/adenosine monophosphate-activated protein kinase in amyotrophic lateral sclerosis patient-derived mesenchymal stem cells can be restored by resveratrol. J. Tissue Eng. Regen. Med..

[205] Kefalakes E., Böselt S., Sarikidi A., Ettcheto M., Bursch F., Naujock M., Stanslowsky N., Schmuck M., Barenys M., Wegner F. (2019). Characterizing the multiple roles of FGF-2 in SOD1 ^G93A^ ALS mice in vivo and in vitro. J. Cell. Physiol..

[206] Müller H.-M., Steringer J.P., Wegehingel S., Bleicken S., Münster M., Dimou E., Unger S., Weidmann G., Andreas H., García-Sáez A.J. (2015). Formation of Disulfide Bridges Drives Oligomerization, Membrane Pore Formation, and Translocation of Fibroblast Growth Factor 2 to Cell Surfaces. J. Biol. Chem..

[207] Thau N., Jungnickel J., Knippenberg S., Ratzka A., Dengler R., Petri S., Grothe C. (2012). Prolonged survival and milder impairment of motor function in the SOD1 ALS mouse model devoid of fibroblast growth factor 2. Neurobiol. Dis..

[208] Sun M., Wang Y., Cheng H., Zhang Q., Ge W., Guo D. (2012). RedoxDB—A curated database for experimentally verified protein oxidative modification. Bioinformatics.

[209] Li Z., Li S., Luo M., Jhong J.-H., Li W., Yao L., Pang Y., Wang Z., Wang R., Ma R. (2022). dbPTM in 2022: An updated database for exploring regulatory networks and functional associations of protein post-translational modifications. Nucleic Acids Res..

[210] Wang P., Zhang Q., Li S., Cheng B., Xue H., Wei Z., Shao T., Liu Z.-X., Cheng H., Wang Z. (2021). iCysMod: An integrative database for protein cysteine modifications in eukaryotes. Brief. Bioinform..

[211] Butterfield D.A., Perluigi M. (2017). Redox Proteomics: A Key Tool for New Insights into Protein Modification with Relevance to Disease. Antioxid. Redox Signal..

[212] Cadenas-Garrido P., Schonvandt-Alarcos A., Herrera-Quintana L., Vázquez-Lorente H., Santamaría-Quiles A., Ruiz de Francisco J., Moya-Escudero M., Martín-Oliva D., Martín-Guerrero S.M., Rodríguez-Santana C. (2024). Using Redox Proteomics to Gain New Insights into Neurodegenerative Disease and Protein Modification. Antioxidants.

[213] Gu L., Robinson R.A.S. (2016). Proteomic approaches to quantify cysteine reversible modifications in aging and neurodegenerative diseases. PROTEOMICS–Clin. Appl..

[214] Zhong Q., Xiao X., Qiu Y., Xu Z., Chen C., Chong B., Zhao X., Hai S., Li S., An Z. (2023). Protein posttranslational modifications in health and diseases: Functions, regulatory mechanisms, and therapeutic implications. Med. Commun..

[215] Rani N., Sahu M., Ambasta R.K., Kumar P. (2024). Triaging between post-translational modification of cell cycle regulators and their therapeutics in neurodegenerative diseases. Ageing Res. Rev..

[216] Paterniti I., Cordaro M., Campolo M., Siracusa R., Cornelius C., Navarra M., Cuzzocrea S., Esposito E. (2014). Neuroprotection by Association of Palmitoylethanolamide with Luteolin in Experimental Alzheimer’s Disease Models: The Control of Neuroinflammation. CNS Neurol. Disord.-Drug Targets.

[217] Assogna M., Casula E.P., Borghi I., Bonnì S., Samà D., Motta C., Di Lorenzo F., D’Acunto A., Porrazzini F., Minei M. (2020). Effects of Palmitoylethanolamide Combined with Luteoline on Frontal Lobe Functions, High Frequency Oscillations, and GABAergic Transmission in Patients with Frontotemporal Dementia. J. Alzheimer’s Dis..

